# Combination therapy targeting Erk1/2 and CDK4/6i in relapsed refractory multiple myeloma

**DOI:** 10.1038/s41375-021-01475-z

**Published:** 2022-01-27

**Authors:** Sophia Adamia, Shruti Bhatt, Kenneth Wen, Zuzana Chyra, Geoffrey G. Fell, Yu-Tzu Tai, Marisa S. Pioso, Ivane Abiatari, Anthony Letai, David M. Dorfman, Teru Hideshima, Kenneth C. Anderson

**Affiliations:** 1 Jerome Lipper Multiple Myeloma Disease Center, Dana-Farber Cancer Institute, Harvard Medical School, Boston, MA 02215 USA; 2 Dana-FArber Cancer Institute, Harvard Medical School, Boston, MA 02215 USA; 3 Department of Pharmacy, National University of Singapore, Singapore, 117559 Singapore; 4 Dana-Farber Cancer Institute, Department of Data science, Boston, MA 02215 USA; 5 Ilia State University, School of Medicine, Tbilisi, G409 Georgia; 6 Department of Pathology, Brigham and Women’s Hospital, Harvard Medical School, Boston, MA 02215 USA

**Keywords:** Targeted therapies, Cancer

## Abstract

Oncogenic activated RAS mutations have been detected in 50% of de novo and 70% of relapsed multiple myeloma (MM) patients. Translocation t(11;14) involving IgH/CCDN1 and overexpression of cyclin-Ds are early events in MM pathogenesis, enhancing uncontrolled MM cell growth. We hypothesized that targeting both *RAS*/*MAPK* pathway molecules including Erk1/2 along with cyclin-Ds enhances MM cytotoxicity and minimizes side effects. Recent studies have demonstrated the high potency of Erk1/2 and CDK4/6 inhibitors in metastatic relapsed cancers, and here we tested anti-MM effects of the Erk1/2 + CDK4/6 inhibitor combination. Our studies showed strong synergistic (IC < 0.5) cytotoxicity of Erk1/2i + CDK4/6i in MM-cells. Erk1/2i + CDK4/6i treatment in a dose-dependent manner arrested MM-cells in the G0/G1 phase and activated mitochondrial apoptotic signaling. Our studies showed that Erk1/2i + CDK4/6i treatment-induced inhibition of key target molecules in Erk1/2 and CDK4/6 signaling, such as c-myc, p-RSK, p-S6, p-RB, and E2F1, suggesting on-target activity of these inhibitors. We identified Erk1/2i + CDK4/6i treatment associated five-gene signature which includes SNRPB and SLC25A5; these genes are involved in RNA processing and mitochondrial metabolism, respectively. Overall, our studies provide the preclinical framework for Erk1/2i + CDK4/6i combination clinical trials to target Ras+CDK pathways to improve patient outcome in MM.

## Introduction

Despite therapeutic advances, MM remains incurable due to the high frequency of relapse and the development of drug resistance [1]. MM relapse and progression are driven by the accumulation of genetic changes within malignant plasma cells (PC) [2–4]. Comprehensive cytogenetic studies in combination with NGS have identified recurrent genetic alterations in MM patients. These alterations include *KRAS*/*NRAS*-activating mutations (>70% relapsed patients), as well as increased CCND1expression due to translocations that involve IGH and a limited set of recurrent partner genes [2–6]. It is known that *KRAS*/*NRAS*-mutations are associated with disease progression, rather than MM initiating events [7]. Dysregulated and increased cyclin-D expressions are early events in MM pathogenesis, enhancing MM cell responses to proliferative stimuli [8, 9]. These findings highlight the therapeutic potential of targeting Ras/Cyclin-D signaling pathways simultaneously in relapsed/refractory MM.

Several MEK- and CDK-inhibitors have been developed and investigated in preclinical and clinical models to target Ras/Cyclin-D pathways in MM, either single agents or in combination with conventional MM therapy [10, 11]. Synergistic growth inhibitory effects were noted when MEK-inhibitors were tested in combination with other anti-MM agents but did not translate to clinical benefit [12–16]. Success in targeting the cell cycle in MM with broad-spectrum CDK-inhibitors has been modest, mainly due to their lack of selectivity and toxicity [17–19]. There are now three FDA approved oral CDK4/6-inhibitors (CDK4/6i) which have shown single agent clinical activity in solid cancers, and in mantle cell lymphoma [20–24]. Recent studies demonstrated high potency of combining novel Erk1/2i (LY3214996) and CDK4/6i (LY2835219) in solid tumors and acute myeloid leukemia, and clinical trials evaluating Erk1/2i + CDK4/6i therapy are ongoing in advanced cancers [20, 24–27].

Targeting of Ras/CDK-dependent pathways remain an attractive strategy in MM since these pathways play essential roles in malignant cell proliferation, cell cycle progression, and deregulation of anti-/pro-apoptotic protein expression associated with resistance to apoptosis in MM-cells. To target Ras/CDK4/6-dependent pathways in MM, we examined Erk1/2i, CDK4/6i, and Erk1/2i + CDK4/6i treatment effects using in vitro and in vivo preclinical models of MM. We demonstrated synergistic cytotoxicity of combination therapy against genetically heterogeneous and drug resistant MM cell lines and patient cells at a broad range of concentrations. We identified the gene-signature associated with response to Erk1/2i + CDK4/6i treatment, which includes *SNRPB* and *SLC25A5* genes. In MM-cells overexpression of these genes might modulate RNA-processing or mitochondrial metabolism, respectively [28–30]. Collectively, our studies provide the preclinical framework for Erk1/2i + CDK4/6i clinical trials to improve MM patient outcomes.

## Materials and methods

### MM cell lines and patients

The human MM-derived cell lines with and without KRAS/NRAS-mutations were purchased from ATCC, and were authenticated within 6 months of use by STR profiling. RAS-wild type (RAS-WT) cell lines carry genetic alterations that induce MAPK pathway activation. MM patient samples were obtained from the MM clinic at Dana-Farber Cancer Institute after obtaining written informed consent.

### Chemical compounds and reagents

Erk1/2i and CDK4/6i were provided by Eli Lilly. Drugs were dissolved in DMSO to 10 mM stock, and drug serial dilutions were prepared for cellular assays, with DMSO concentration not exceeding 0.1%.

### Cell proliferation studies

Trypan blue exclusion assay was utilized for cell counting prior to seeding cells for proliferation studies using CellTiter-Glo assay, according to manufacturer’s instructions (Promega). In proliferation assays, the drug concentration that caused 50% inhibition of cell proliferation relative to DMSO control was determined by non-linear regression using Prism-8.

### Drug combination studies

For drug combination studies, single drugs were added simultaneously at fixed ratios to cells. Cell viability was expressed as a function of growth affected in drug-treated versus DMSO control cells, followed by data analyses by Calcusyn software.

### Apoptosis assays and cell cycle analysis

Drug-induced apoptosis was assessed using the Annexin-V FITC and CellEvent Caspase-3/7 Green, according to manufacturer’s instructions (ThermoFisher). Cell cycle analysis was performed as previously described [27].

### Protein expression

Protein lysate preparation and immunoblotting analyses were carried out as previously described [31]. Antibodies were purchased from Cell Signaling Technology and used according to manufacturer’s instructions (Supplementary Material S1 (SM1)). InstantOne ELISA Kits were used to evaluate total and phosphorylated Erk1/2 levels.

### Dynamic BH3 profiling

MM cell lines were exposed to a panel of targeted agents or DMSO (control), followed by BH3 profiling using BH3 peptides, as previously described (SM2) [32].

### Non-invasive in vivo bioluminescence study

Animal studies were performed according to protocols approved by the Dana-Farber Cancer Institute’s Institutional Animal Care and Use Committee. Bioluminescence imaging was performed as described in SM3.

### RNA-seq and identification of treatment related molecular signature

RNA seq analyses and validation studies were carried out as described in SM4.

## Results

### ERK1/2 and CDK4/6 are activated and overexpressed in MM

**Fig. 1:**
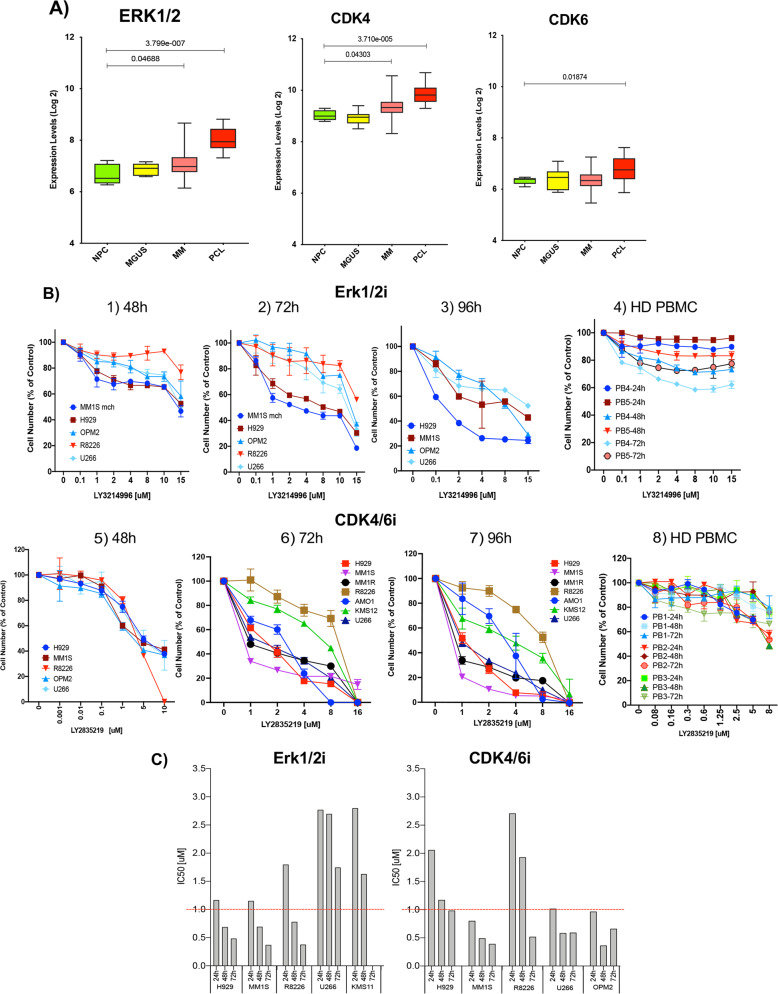
Effects of Erk1/2i + CDK4/6i on MM cell lines and HD-PBMC. **A** Box plots showing *ERK1/2* and *CDK4/6* gene expression values in CD138 + PC from monoclonal gammopathy of undetermined significance (MGUS; *n* = 11), multiple myeloma (MM; *n* = 133), plasma cell leukemia (*n* = 9; PCL), and NPC (healthy donors), from dataset GSE13591. The error bars show the standard deviation. Horizontal bars indicate the mean value. The x-axis shows the samples analyzed, and the *y*-axis displays the expressions at log2 fold. These analyses confirmed significant (0.05 > *P* < 0.3E-0.07) overexpression of *ERK1/2, CDK4 and CDK6* transcripts in clonal PCs. **B** Efficacy studies in MM cell lines (H929, MM.1Smch (mCherry (red fluorescent protein)), MM1R, AMO1, KMS12, RPMI8226, OPM2, and U266) and HD PBMCs at different time points after LY3214996 (Erk1/2i) and LY2835219 (CDK4/6i) treatment at different concentration ranges are shown on the *x*-axis; Cell viability (means and standard deviations) is shown as percentage of control untreated cells. **C** Determination inhibitory potency (IC50) of Erk1/2i for MM. MM (H929-NRAS, MM.1S, RPMI8226. OPM2 and U266) cell lines were treated with DMSO or ERK1/2i (0-15uM MM cell lines in RPMI medium with 0.1% DMSO and 10% FBS for 24–72 h. The concentration of drug that caused 50% inhibition of cell proliferation (IC50) relative to DMSO control was determined by non-linear regression using Prism, version 8.

We assessed expression of *ERK1/2* and *CDK4/6* transcripts in MM patient cohort (GSE13591-GSE2658-GSE5900). We confirmed increased expression of *ERK1/2 (in MM P* < 0.05 or 0.04; in plasma cell leukemia (PCL), *P* < 0.3E-0.07) and *CDK4/6 (P* < 3.7E-005 or 0.0006; *P* < 0.02 or 0.05) in clonal PCs with progression from monoclonal gammopathy of undetermined significance *(*MGUS) to MM and PCL compared to healthy donor PC (NPC) (Fig. 1A, Fig. S1A). Activation and overexpression of p-Erk1/2 and CDK4/6 and their involvement in MM pathophysiology and clinical significance have been extensively studied by our and other groups [14, 15, 33–35]. These data collectively suggest the potential clinical benefits of targeting these pathway molecules simultaneously.

### Synergistic effects of ERK1/2i and CDK4/6i in MM

Before evaluation synergistic effects of ERK1/2i + CDK4/6i treatment, we first evaluated antiproliferative effects of Erk1/2i and CDK4/6i as single agents. Both *RAS-*MT and WT MM cell lines were treated with DMSO or Erk1/2i/CDK4/6i at increasing concentrations at different time points (Fig. 1B, C, Fig. S1B–D,S2). The growth of *NRAS* or *KRAS* cell lines was inhibited after 48 h with IC50 of ~0.7 μM, while *RAS-*WT cell, growth was inhibited at IC50 of 2.7 μM and 1.7 μM, respectively. After 72 h and 96 h of treatment, the sensitivity of all MM cell lines to Erk1/2i increased, with IC50 of 0.1–0.5 μM in *RAS*-MT cells and IC50 of 1–3 μM in WT cells (Fig. 1B2–3C, Fig. S1D). Some of the RAS-WT cell lines carry a BRAF activating mutation or t(11;14) and t(4;14) alterations. Significant growth-inhibition was seen in all MM cell lines after 72 h treatment with CDK4/6i (IC50 0.4–2 μM): 80% cell-death was noted in both *RAS*-WT and *RAS*-MT cells (*P* < 0.005) (Fig. 1B6, Fig. S2). In contrast, HD (healthy donor) PBMC were relatively resistant to Erk1/2i (0–15 μM for 72 h) and CDK4/6i (0–2.5 μM for >96 h), suggesting a favorable therapeutic index (Fig. 1B4,B8, Fig. S2). Collectively, Erk1/2i studies show IC50 of 0.1–1 μM for *RAS-*MT and 1–3 μM for *RAS*-WT MM lines (Fig. 1C).

Our prior studies show that BM stromal cells (BMSC) activate RAS/RAF/MAPK signaling in MM, triggering MM cell growth and drug resistance [12, 14, 15, 35–39]. We therefore next evaluated whether Erk1/2i can overcome this protective effect of MM BMSC. MM cell lines with (MM1S and H929) and without (U266, OPM2) *RAS* mutations were treated with Erk1/2i (0–16 μM for 24 h, 48 h, 72 h, 96 h) in the presence or absence of MM BMSC-conditioned media (BMSC-CM) (Fig. S1E). IC50 of ~1 μM was observed in *NRAS*-MT H929 cells after 48 h culture with MM BMSC-CM; was more delayed (96 h) in *KRAS*-MT MM1S cells. Growth-inhibition by Erk1/2i was detected in U266 or OPM2 cell lines carrying a BRAF activating mutation and t(11;14), or t(4;14) (IC50 3–5 μM).

**Fig. 2:**
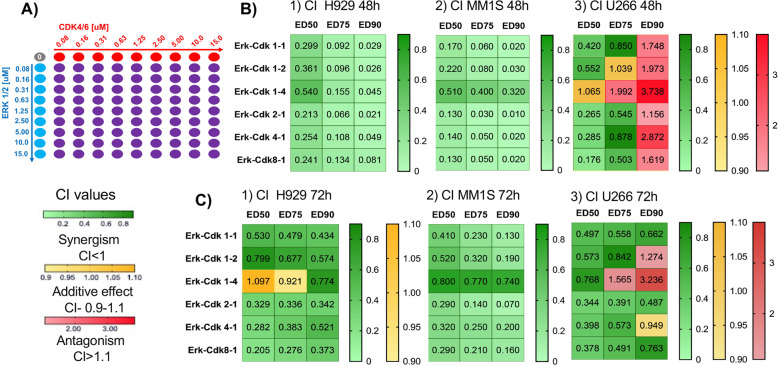
Synergistic cytotoxic effects of Erk1/2i + CDK4/6i on MM-cells. **A** Schematic diagram of 9×9 checker-board matrix of ERK1/2i and CDK4/6i inhibitor concentrations in cell viability assays performed at 48 h (**B**) and 72 h (**C**) in MM1S, H929, and U266 cells co-cultured with BMSC-conditioned medium. CalcuSyn software was used to assess the synergistic effects of ERK1/2i + CDK4/6i. Calcusyn combination index (CI) is interpreted as follows: CI < 0.8 indicates synergism; CI < 0.55 indicates strong synergism; 0.6 < CI < 0.8 indicates moderate synergism; 0.9 < CI < 1.1 indicates additive effects; 1.2 < CI < 1.9 indicates moderate antagonistic effects. ED50, ED75, ED90 = effective dose at 50%, 75%, and 90%, respectively. H929-NRAS, MM1S-KRAS, and U266 WT cells were treated with ERK1/2i and CDK4/6i. These data demonstrate highly potent cytotoxicity of Erk1/2i + CDK4/6i combination 48 h after treatment. This highly synergistic effect of Erk1/2i + CDK4/6i was retained 72 h after treatment.

We used the checker-board method to assess the effect of Erk1/2i + CDK4/6i treatment on H929-NRAS, MM1S-KRAS, and U266 WT MM-cells co-cultured for 48 h and 72 h with MM-BMSC-CM (Fig. 2A). After 48 h treatment with Erk1/2i + CDK4/6i, we observed synergistic growth-inhibition in mutant-*RAS* expressing cells (MM1S/H929), with a combination index (CI) < 1 across a broad range of concentrations (Fig. 2B1, 2). After 72 h, Erk1/2i + CDK4/6i treatment of MM1S/H929 cells remained strongly synergistic (CI < 0.5) at the majority of drug combination doses (Fig. 2C1, 2). We observed more potent synergistic activity of Erk1/2i + CDK4/6i in U266 cells after 72 h treatment compared to 48 h (Fig. 2B3, C3). Collectively, these data demonstrate highly potent MM cytotoxicity in both *RAS*-MT (H929 and MM1S) and *RAS*-WT (U266) MM-cells when Erk1/2i is combined with CDK4/6i. The similar Erk1/2i + CDK4/6i treatment effects in both *RAS*-MT and *RAS*-WT MM-cells can be due to the fact that RAS-WT U266 cells carry BRAF activated mutation in combination with a t(11;14). Our study agrees with the recent study which demonstrates that MAPK-pathway activity in MM is a result of the combined effects of RAS-mutations and various chromosomal alterations while considering the effects of the microenvironment [40].

### Erk2i + CDK4/6i trigger cell cycle arrest and induce mitochondrial-dependent apoptosis

**Fig. 3:**
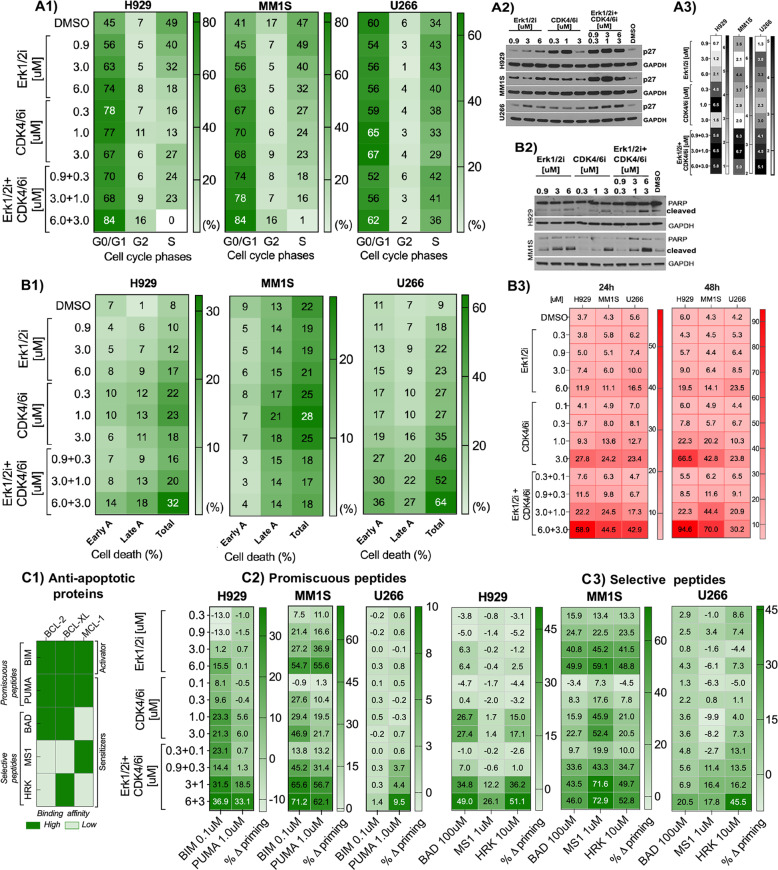
Erk1/2i + CDK4/6i treatment triggers cell-cycle arrest and induces apoptosis. Erk1/2i + CDK4/6i induced cellular effects were evaluated in H929, MM 1S, and U266 cells co-cultured with BMSC-conditioned medium after 48 h treatment with ERK1/2i (0; 0.9; 3 and 6 μM) and CDK4/6i (0; 0.1; 1 and 3uM), alone or in combination. **A1** Cell cycle profiles were analyzed using standard DAPI staining. MM-cells in G0/G1, S, and G2/M phases were measured on the BD Fortessa X-20, followed by analysis using ModFit LT software. **B1** ERK1/2i + CDK4/6i effects on apoptosis were quantified using annexin V-fluorescein isothiocyanate and 7-AAD staining and flow cytometry; percentages of early apoptotic (early A; Annexin V+/7-AAD−) and late apoptotic (late A, Annexin V+/7-AAD+) events were identified using FlowJo V10 software. **A2** and **B2** Protein lysates were obtained after treatment of MM-cells with Erk1/2i or CDK4/6i or Erk1/2i + CDK4/6i. Cell lysates were subjected to immunoblotting using the antibodies indicated; GAPDH served as a loading control. **A3** Densitometry analysis of protein bands was performed using the Image J software. The color key next to each heatmap shows the protein expression level compared to DMSO. On the heatmaps, protein expression levels are indicated with intensity shades of black color. These analyses showed a dose-dependent overexpression of a cell cycle inhibitor p27 protein (**A2**), and increased PARP cleavage (**B2**), the signature of cell death. **B3** Caspase (CAS) 3/7 activation in MM cell lines was measured by flow cytometry in response to Erk1/2i, CDK4/6i, and Erk1/2i + CDK4/6i treatment. Relative CAS3/7 activation is summarized as heatmaps; mean fluorescent intensities (MFI) are calculated in comparison with MFI of controls. Each treatment with a specific concentration of Erk1/2i and/or CDK4/6i was done in duplicate, plus/minus the standard error of the mean has been hidden for simplicity. **C** BH3 profiling to measure early changes in net pro-apoptotic signaling of mitochondria in response to ERK1/2i and CDK4/6i, alone or in combination. **C1** Interaction map for BH3 peptides and BH3 mimetics with BCL-2 family proteins. Darker green color indicates Kd<100 nM determined by fluorescence polarization. **C2, C3** Heatmap of delta priming response, assessed by dynamic BH3 profiling, in MM cell lines plated and treated for 16 h with ERK1/2i and CDK4/6i, alone or combination. Delta priming = % cytochrome *c* loss_(drug)_ − % cytochrome *c* loss _(DMSO)_. **A, B, C** Results are summarized as heatmaps; color-keys are shown next to the each heatmap for data interpretation. Expression levels are indicated with intensity shades of green color.

To address the underlying mechanism of the synergism, we evaluated the impact Erk1/2i/CDK4/6i on cell cycle profile and apoptosis in MM cells. These cells were treated with Erk1/2i + CDK4/6i combinations that were strongly synergistic (Fig. 2B, C, CI < 0.5). Treatment of MM1S and H929 cells with ERK1/2i + CDK4/6i arrested cells in the G0/G1 phase in a dose-dependent manner, evidenced by an increase percentage (~40%) of cells in the G1-phase, and a decreased percentage (~20–40%) of cells in S-phase (Fig. 3A1). A dose-dependent cell-cycle arrest in MM-cells treated with Erk1/2i + CDK4/6i is evidenced by increased expression of the CDK inhibitor protein p27 (Fig. 3A2–3). There was no significant change observed in cell-cycle arrest in U266 cells.

We evaluated the effect of ERK1/2i + CDK4/6i on apoptosis. Annexin V-AAD staining analysis revealed that Erk1/2i and CDK4/6i (alone or in combination) in a dose-dependent manner induced 22–32% apoptosis in *RAS*-MT MM1S and H929 cells (Fig. 3B1). In U266 cells without *RAS* mutations, but with a BRAF activating mutation and t(11;14), ERK1/2i and CDK4/6i triggered 22–60% apoptosis. At the protein level, dose-dependent increased expression of cleaved-PARP and CAS3/7 were observed in MM cells (Fig. 3B2-3). These effects were most pronounced in cells treated with Erk1/2i + CDK4/6i at concentration ratios of 3:1.

Activated Erk1/2 and CDK4/6 can access specific substrates into the mitochondria and consequently “force” sensitization of malignant cells to undergo apoptosis [41, 42]. To evaluate whether Erk1/2i + CDK4/6i triggers mitochondria-dependent apoptosis, we performed dynamic BH3 profiling (DBP) after treatment. Specifically, we measured cytochrome c release triggered by BIM or PUMA peptides as a metric of apoptotic sensitivity or a priming of MM-cells for apoptosis (Fig. 3C1). Treatment with ERK1/2i and CDK4/6i, alone or in combination, induced dose-dependent delta priming to PUMA and BIM peptides in MM-cells. Cell treatment with Erk1/2i or CDK4/6i, respectively, induced 55% and 46% priming to the BIM peptide in MM1S cells, and 16% and 23% priming to this peptide in H929 cells (Fig. 3C2). Treatment with Erk1/2i + CDK4/6i combination induced dose-dependent increased delta priming (10–62%) to the PUMA peptide (Fig. 3C2) in all cell lines compared to the DMSO control. Overall, this effect was more pronounced in MM1S and H929 cells, relative to U226 cells.

To better characterize the mechanism of apoptotic priming in MM-cells, we used three selective peptides: BAD, MS1, and HRK. These peptides detect dependencies on BCL2/BCLXL, MCL1, or BCLXL. The Erk1/2i + CDK4/6i treatment enhanced the mitochondrial priming response to BAD and HRK peptides, in all cell lines, indicating a survival dependence of these cells on BCL-2/BCL-XL (Fig. 3C3). MM1S cells showed a co-dependence on MCL-1, as indicated by an increased sensitivity to the MS1 peptide (43–73% priming). Our results suggest that ERK1/2i and CDK4/6i, alone or in combination, activate pronounced mitochondrial apoptotic signaling in all MM cell lines tested. This effect was more pronounced in MM1S and H929 cells, relative to U266 cells; this is consistent with their synergistic cytotoxicity induced in these cells by ERK1/2i + CDK4/6i treatment (Fig. 2B, C).

### Erk1/2i + CDK4/6i selectively targets Erk1/2 and CDK4/6 and their downstream substrates

**Fig. 4:**
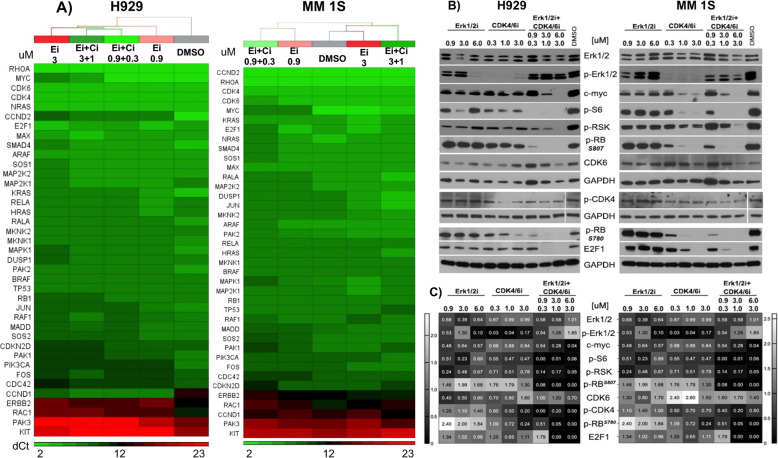
Effects of ERK1/2i + CDK4/6i on gene and protein expression in MM cell lines. H929, MM 1S, and U266 cells co-cultured with BMSCs were treated for 48 h with ERK1/2i (Ei) (0; 0.9; 3 and 6 μM) and CDK4/6i (Ci) (0; 0.1, 1, and 3 μM), alone or combination (Ei + Ci). MM-cells were then separated from BMSC using EasySep magnetic bead cell separation method. **A** Total mRNAs were isolated from MM1S and H929 cells and transcribed into cDNAs. Gene expression profiling was performed using custom TaqMan Gene Expression Array Plates containing primers and probes for detection of RAS and cell cycle signaling pathway genes. Results were analyzed using the relative standard curve method, and final data analyses and heatmaps were generated based on dCt values using Partek Genomic Suite. Expression levels are indicated with intensity shades of green/red colors. GAPDH and TBP genes were used as housekeeping genes. **B** Protein lysates (20–40 μg of protein/lane) of H929 and MM1S cells were separated on SDS-PAGE, and after blotting onto nitrocellulose membranes were probed with anti- Erk1/2, -p-ERK1/2, -c-myc, -p-S6, p-RSK, -p-RB, and -E2F1 antibodies; GAPDH served as a loading control. **C** Densitometry analysis of protein bands was performed using the Image J software. The color key next to each heatmap shows the protein expression level compared to DMSO. On the heatmaps, protein expression levels are indicated with intensity shades of black color.

To characterize the functional effects of ERK1/2i + CDK4/6i on their downstream target substrates and to further delineate the molecular mechanism underlying the synergism between Erk1/2i and CDK4/6i, we evaluated transcriptional/proteomic changes triggered by treatment with Erk1/2i, CDK4/6i or combinations. Gene expression profiling showed downregulation of both Ras and CDK4/6 signaling pathway genes in MM-cells after ERK1/2i + CDK4/6i at concentration ratios of 3:1 or 2:1, which are associated with synergistic cytotoxicity (Figs. 4A, S3, 2B, C). We also monitored effects of these treatments at the protein level: drug-induced transcript inhibition correlated with inhibition of total Erk1/2, p-Erk1/2, and CDK levels, and key targets c-myc, p-RSK, p-S6, p-RB, and E2F1, confirming their on-target functional activity (Fig. 4B, C).

**Fig. 5:**
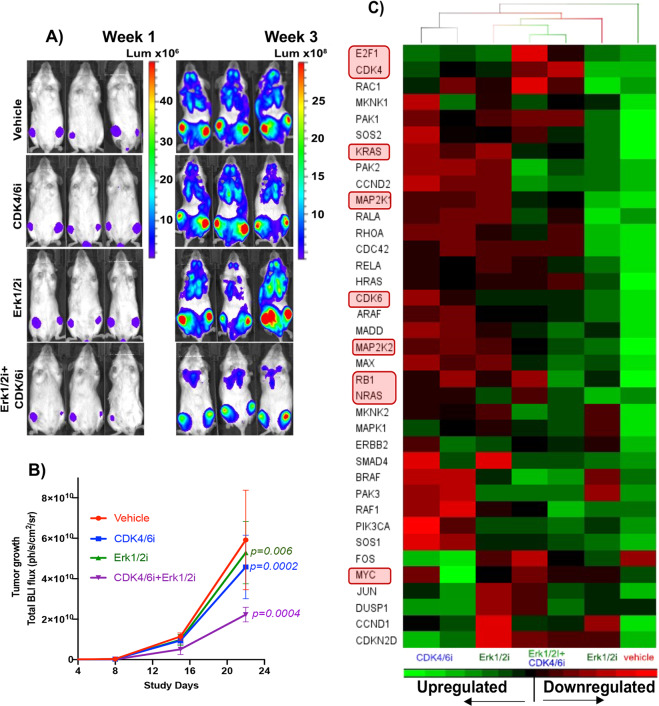
Erk1/2i + CDK4/6i reduces tumor burden in an MM1.S-Luc^+^ xenograft in vivo model. **A** We specifically used this model in which MM1S cells home to the mouse BM, mimicking the tumor microenvironment of human MM. BLI images of three representative mice bearing MM1.S-Luc^+^ tumors from each indicated group. BLI images showing localization MM1.S-Luc^+^ tumor cells to hind limb BM. The scale represents luminescence signal from MM1.S-Luc^+^ cells**. B** BLI imaging quantification of tumor burden for monitoring tumor progression; data are presented as mean values ± SD (*n* = 7-8 animal/group); P values were calculated using Log-rank (Mantel-Cox) test in Prism V9. **C** Total mRNAs were isolated from bone marrow cells obtained from flushed femurs of animals treated with Erk1/2i (Ei) or CDK4/6i (Ci), alone or combination (Ei + Ci); then these samples were transcribed into cDNAs and subjected to the RAS and cell cycle signaling pathway gene-expression profiling using custom TaqMan Gene Expression Arrays. Each of the TaqMan assays was individually evaluated as a single reaction. The TaqMan assays that specifically recognize human mRNA (polyadenylated transcripts encoding proteins) were included in the final screening arrays. Results were analyzed using the relative standard curve method and presented as heatmaps (Partek Genomic Suite). Gene-expression levels (dCt values) are indicated with green/red color intensity. GAPDH/TBP was used as a housekeeping gene. These analyses showed significant downregulation of RAS and CDK4/6 signaling pathway genes in samples treated with Erk1/2i + CDK4/6i.

We next explored whether in vitro anti-MM activity of Erk1/2i + CDK4/6i could be translated in vivo to a disseminated MM murine model. As shown in Fig. 5A, B, tumor burden in mice treated daily with Erk1/2i + CDK4/6i for 4 weeks was significantly (*P* = 0.0004) reduced compared with animals treated with either the inhibitor alone or DMSO control. Importantly, the Erk1/2i or CDK4/6i treatment was well tolerated, with no significant change in the body weight of Erk1/2i + CDK4/6i treated mice suggesting a favorable therapeutic index.

We also evaluated the effect of ERK1/2i + CDK4/6i on transcriptional activity of target genes in BM cells isolated from flushed femurs of animals treated with ERK1/2i, CDK4/6i or ERK1/2i + CDK4/6i. We observed downregulation of Erk1/2- and CDK4/6-dependent transcriptional gene signature (Fig. 5C). These analyses are consistent with our in vitro analyses done on MM cell lines. Our studies suggest that these inhibitors are selectively targeting Erk1/2 and CDK4/6 and their downstream substrates both in vitro and in vivo.

### Evaluation of potential clinical utility of Erk1/2i + CDK4/6i treatment in MM

**Fig. 6:**
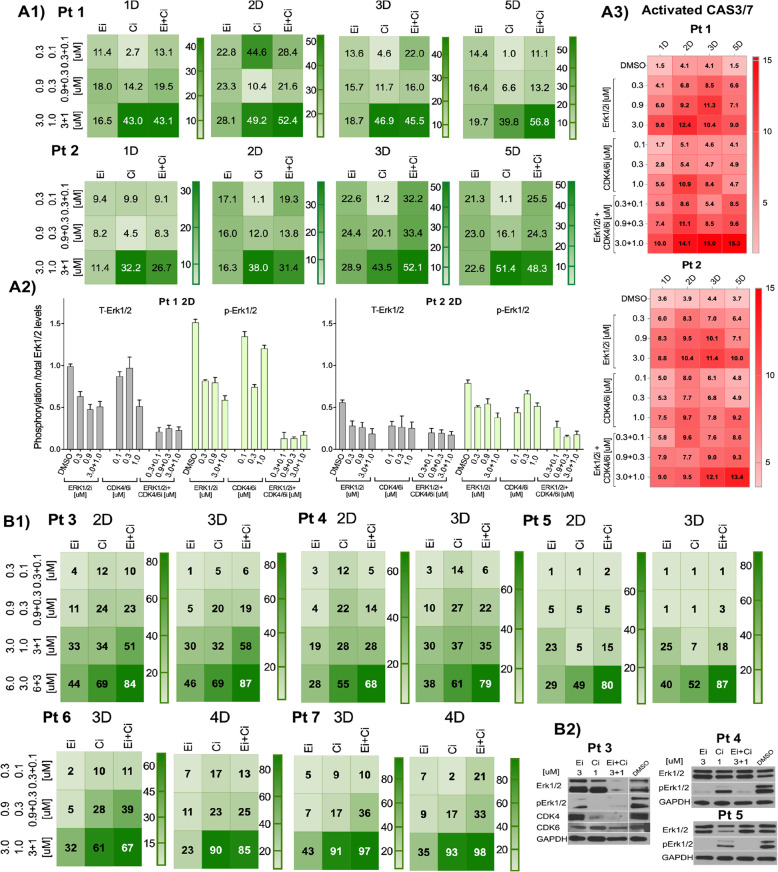
Effects of Erk1/2i and CDK4/6i on primary MM-cells (A 1, B1, B3). Dose-response effects of Erk1/2i (Ei), CDK4/6i (Ci), or Erk1/2i + CDK4/6i (Ei + Ci) treatment on MM-cells freshly obtained from MM BM aspirates of patients are summarized as heatmaps. CD138 + PCs selected from MM patient BM using EasySep cell separation, with purity >95% (confirmed using flow cytometry), were treated with Erk1/2i, CDK4/6i, or Erk1/2i + CDK4/6i at the concentrations indicated. On the figures **A1**, **B1** the results are shown as percent (%) of cell death relative to DMSO controls. The % cell death is indicated with intensity shades of green color scale shown at the right edge of each heatmap. Each concentration of Erk1/2i and/or CDK4/6i was tested in triplicate. The results, plus or minus the standard error of the mean, ranged from +0.01 to +0.7, and have been hidden for simplicity. **A2** CD138 + PC were collected 48 h after treatment and cell lysates were prepared to measure phosphorylation/total levels of Erk1/2 using InstantOne enzyme-linked immunosorbent assay kits, according to the manufacturer’s protocol. Absorbance was measured at 450 nm. The results are presented as bar graphs. The *x*-axis shows the samples analyzed, and the *y*-axis displays the phosphorylation/total Erk1/2 levels as an absorbance. Results obtained from positive and negative control samples are not displayed on the graph. The mean ± S.D. is shown for two independent experiments. **A3** Caspase (CAS) 3/7 activation in MM CD138 + PCs from Pt 1 and Pt 2 were measured by flow cytometry in response to ERK1/2i and CDK4/6i treatment, alone or in combination. In this experiment, unfractionated BM cells from MM patients were used, and CAS3/7 activation was measured in MM CD138 gated PCs. The results are summarized as heatmaps; the percentage of mean fluorescent intensity (MFI) was calculated and compared with the MFI of the controls, and presented in shades of red scale shown on the right side of each heatmap. Each treatment with a specific concentration of Erk1/2i and/or CDK4/6i was done in duplicate, plus/minus standard error (+0.01 to +0.75) of the mean MFI have been hidden for simplicity. **B2** Functional effects of ERK1/2i and CDK4/6i or ERK1/2i + CDK4/6i treatments were validated at the protein level by immunoblotting. Cell lysates were prepared 2D after treatment from CD138 + PCs selected using a CD138 positive magnetic bead selection method (Stem Cell Technology).

To further assess the potential clinical utility of ERK1/2i + CDK4/6i, we next evaluated the effects of these inhibitors on MM-cells obtained from patients with relapsed/ refractory disease. Similar to MM cell lines, 15–53% cytotoxicity was detected in patient cells treated for 2D with Erk1/2i + CDK4/6i combination (ratio 3:1) (Fig. 6A1, B1). MM cell cytotoxicity increased 3D after treatment, and in some samples reached 85% (Fig. 6A1–B1, 7A). Importantly, treatment with Erk1/2i + CDK4/6i led to concentration-dependent synergistic (CI 0.3–1) MM cell death.

**Fig. 7:**
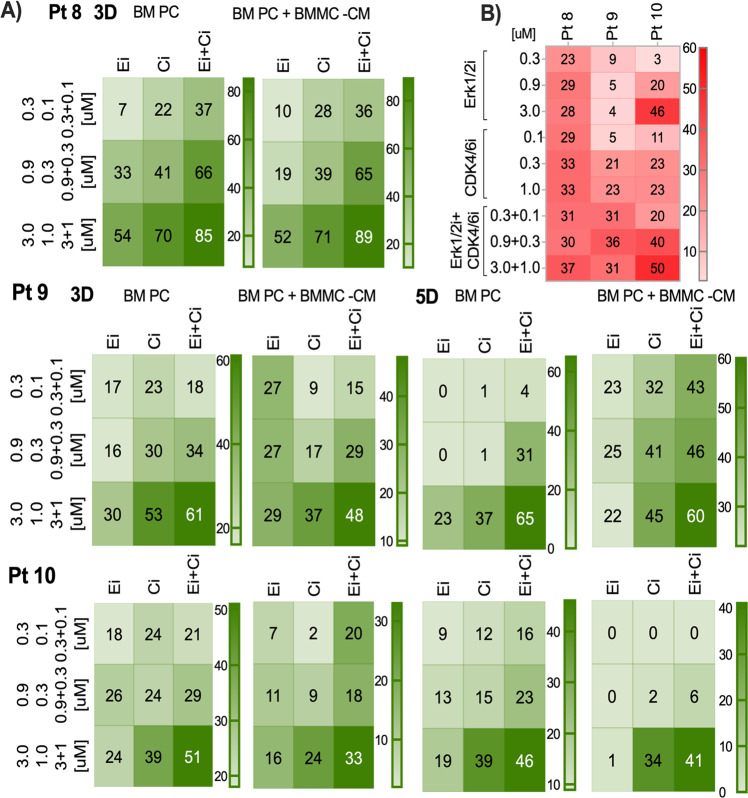
Effects of ERK1/2i + CDK4/6i on primary MM-cells co-cultured with or without BMSC-CM. This cohort includes two patients with NRAS mutations, three patients with KRAS mutations, five patients with RAS-WT, and two patients with the t(1;14). **A** CD138 + PCs were cultured with or without BMSC-CM and treated for 3D and 5D. MM PCs were maintained in 15% fetal calf serum spiked with the serum obtained from autologous peripheral blood of MM patients. Cells were treated with Erk1/2i (Ei), CDK4/6i (Ci), or Erk1/2i + CDK4/6i (Ei + Ci) at the concentrations indicated. The % cell death is indicated with intensity shades of green colors, and the color scale is shown at the right edge of each heatmap. Each concentration of Erk1/2i and/or CDK4/6i was tested in triplicate. The results, plus or minus the standard error of the mean, ranged from +0.01 to +0.7. **B** Erk1/2i, CDK4/6i, or Erk1/2i + CDK4/6i effects on apoptosis in MM BM PCs were quantified using Annexin V + 7-AAD staining. Unfractionated BM cells were treated at the indicated concentrations, and cell death was monitored 3D after treatment; % of apoptotic events were detected in CD138 gated PCs using FlowJo V10 software. Flow cytometry data that illustrates the gating strategy for the apoptosis assay is shown in supplementary Fig. S5.

Functional effects of ERK1/2i and CDK4/6i or ERK1/2i + CDK4/6i treatments were validated at the protein level by the InstantOne enzyme-linked immunosorbent assay or immunoblotting (Fig. 6A2–B2). Consistent with the observed therapeutic effect, analyses of patient BM samples confirmed that 2D treatment with Erk1/2i + CDK4/6i significantly decreased total Erk1/2, and p-Erk1/2 and/or CDK4 levels (Fig. 6A2–B2). The combination treatment induced increased apoptotic patient MM cell death, evidenced by the abundance of cleaved-CAS3/7 levels and by Annexin-V/7-AAD (Fig. 6A3, 7B). Importantly, 30–50% drug-induced MM cytotoxicity was observed after 3D treatment with Erk1/2i + CDK4/6i in the presence/absence of autologous MM BMSC-CMs, indicating that this combination may overcome the protective effect of the MM-BM-milieu (Fig. 7A).

### Gene signature-based approach identified targets associated with Erk1/2i + CDK4/6i

**Fig. 8:**
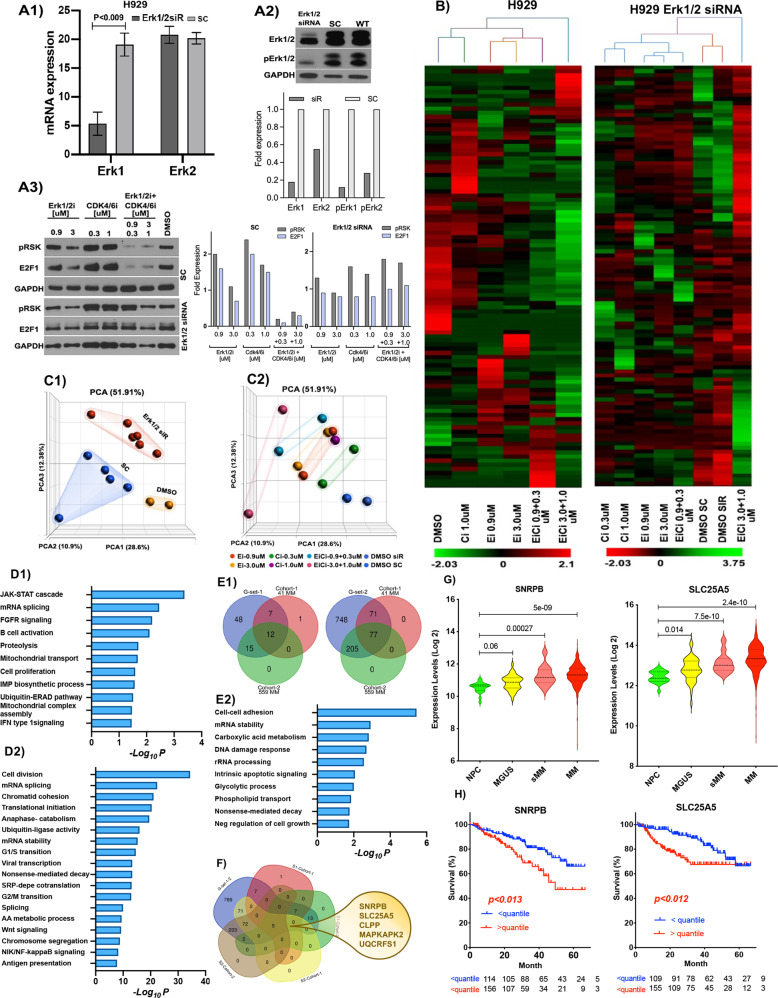
Gene signature-based approach identified targets associated with Erk1/2i + CDK4/6i treatment. **A** H929 MM-cells were transfected with either scrambled control (SC) or Erk1/2-siRNA (Erk1/2-siR); Erk1/2 knockdown efficiency was evaluated at mRNA (**A1**) and at protein (**A2**) levels. Erk1/2 knockdown H929 cells were treated with Erk1/2i, CDK4/6i, or Erk1/2i + CDK4/6i; after 48 h of treatment cell lysates were collected **(A3)**. Protein lysates **A2, A3** were subjected to immunoblotting using Erk1/2, p-ERK1/2, pRSK, and E2F1 antibodies; GAPDH served as a loading control. Bar graphs in **A2** and **A3** show a densitometric analysis of the protein bands measured by ImageJ software. Fold expressions on the *Y*-axis show the protein expression level compared to loading controls. **B** A heatmap shows unsupervised clustering of deregulated genes detected in H929 cells, with or without Erk1/2 knockdown or treated with Erk1/2i and CDK4/6i, alone or in combinations. The color scale for log expression values is shown at the bottom of the heatmaps, while sample clustering is presented as a dendrogram on the top. **C** Transcriptome changes in these cells are shown by principal component analysis (PCA). On PCA plot C1, different groups are presented in different colors; on PCA plot C2, cells treated with different concentrations of the drugs or drug combinations are presented with different colors. **D** Gene and functional enrichment analyses of Erk1/2i + CDK4/6i signature gene-sets (G-set-1 (**D1**) and G-set-2 (**D2**)) were performed using DAVID v6.8. G-set-1 comprises the genes that are upregulated in response to Erk1/2 knockdown (G-set-1); G-set-2 includes genes that were downregulated in response to Erk1/2i + CDK4/6i treatment. Enrichment analyses considered p-value enrichment with *p* < 0.05, and fold enrichment with a *p* > 5. Bar plots display the −*log10 p* value enrichments. **E1** The Venn method was used to identify commonly deregulated signature genes associated with Erk1/2i + CDK4/6i treatment. The left Venn diagram–intersecting deregulated G-set-1 with deregulated genes in MM Cohort-1(GSD4968), and Cohort-2(GSE5900, GSE2658); the right Venn diagram-intersecting deregulated G-set-2 with deregulated genes in MM Cohort-1/2. **E2** Gene and functional enrichment analyses of commonly deregulated Erk1/2i + CDK4/6i signature genes identified in MM cohort-1/2. Enrichment analyses considered *p* value enrichment with *p* < 0.05, and fold enrichment with a *p* > 5. Bar plots display the −log10 *p* value enrichments. **F** Identification five gene-signature associated with Erk1/2i + CDK4/6i. The Venn diagram–intersecting deregulated G-set-1and G-set-2 with deregulated genes in MM Cohort-1/2. **G** Violin plots showing gene expression values in CD138 + PC from MGUS (*n* = 44), sMM (*n* = 12), MM (*n* = 559), and HD (healthy donor, *n* = 22), from GSE5900 and GSE2658. The *x*-axis shows the samples analyzed; violin plots are colored by sample type. The *y*-axis displays the expressions at log2 fold. Significance between groups was evaluated using a Wilcoxon Rank-Sum test; the type I error cut off was 5%. Multiple testing adjustments between groups were then made using the Bonferroni correction. These analyses show significant overexpression of *SNRPB and SLC25A5* transcripts in clonal PCs. **H** The relevance of *SNRPB and SLC25A5* expression to clinical outcome was examined in 559 MM patient samples. The samples were classified based on *SNRPB* and *SLC25A5* expression levels. Survival curves were estimated using the Kaplan-Meier method; the type I error cut off was 5%. differences in survival were assessed using the log-rank test and Cox regression models. For analyses R version 4.0.0, along with the survival and survminer packages, were used. >quantile = >75th percentile; <quantile = <25th percentile.

To identify a molecular signature associated with the Erk1/2 + CDK4/6i treatment, we evaluated transcriptome changes in H929 cells transfected with ERK1/2 siRNA followed by treatment with Erk1/2i and CDK4/6i, alone or in combination. We then carried out RNA-seq analyses of the samples (Fig. 8). We verified the Erk1/2 knockdown efficiency at mRNA and protein levels (Fig. 8A1, A2). As Fig. 8A1 indicates, Erk1/2 siRNA achieved 72% downregulation of total Erk1, while total Erk2 transcript levels did not change. Nevertheless, protein level analyses showed downregulation of total Erk1 (82%) and Erk2 (45%) expressions and significantly decreased p-Erk1(82%) and p-Erk2 (72%) levels, in siRNA transfected cells (Fig. 8A2). The levels of p-RSK and E2F1, which are key targets of the Erk1/2 and CDK4/6 pathways, did not change in siRNA transfected cells, while levels of these targets changed dose-dependently in cells transfected with the SC (Fig. 8A3). These results suggest the on-target activity of Erk1/2i on MM-cells.

RNAs were next extracted from knockdown or treated cells and subjected to RNA-seq. Unsupervised clustering analyses of deregulated (up or down-regulated) genes detected in these cells showed that cells treated with Erk1/2i or CDK4/6i cluster together under first-degree sub-nodes, suggesting close similarities between deregulated gene signatures (Fig. 8B). However, samples treated with Erk1/2i + CDK4/6i combination clustered as a second-degree sub-node of the samples treated with a single agent, suggesting that samples treated with Erk1/2i + CDK4/6i express related but distinct deregulated gene-sets. This observation was supported by principal component analyses (PCA), which showed a separation between treated groups and control samples (Fig. 8C1). PCA analyses demonstrated dose-dependent treatment effects as well (Fig. 8C2). Samples treated with a higher dose of Erk1/2i + CDK4/6i are even further segregated from the control group, indicating selective effects of this combination on the MM transcriptome.

Next, we focused on the genes that were deregulated in response to Erk1/2 knockdown (G-set-1) and downregulated in Erk1/2i + CDK4/6i treated cells (G-set-2) (Table S1A, S2A). We consider these gene-sets as a signature associated with Erk1/2i + CDK4/6i treatment. Gene and pathway enrichment analyses of G-set-1/2 identified several Erk1/2i + CDK4/6i targets that are implicated in MM parthenogenesis (FGFR3, BCMA, IRF4, and GABARAP) (Fig. 8D1, 2). These analyses identified Erk1/2i + CDK4/6i treatment-associated genes, including *RAVER1* and *SNRPB*. These genes encode subunits of the U1-spliceosome complex and are involved in altered RNA splicing specifically in the intron retention process, which is a unique marker of malignant transformation [43, 44]. We validated Erk1/2i + CDK4/6i signature-gene (G-set-1/2) expression in two independent MM patient cohorts (GDS4968 and GSE5900-2658), using previously described data analyses pipeline (Table S3) [45]. Compared to HD PCs, we identified significant (*p* < 0.001) deregulation of 12 genes of G-set-1 and 77 genes of G-set-2 in patient MM-cells (Fig. 8E1, Table S4). Gene enrichment/functional annotation analyses of these genes showed their involvement in cell proliferation and regulation of epigenetic networks in MM (Fig. 8E2). Using the Venn method, we identified a commonly deregulated gene signature (*SNRPB, MAPKAPK2, SLC25A5, CLPP*, and *UQCRFS1*) associated with Erk1/2i + CDK4/6i treatment (Fig. 8F). We validated the expression of these signature genes compared to NPC in MM cells from 558 MM patient samples and observed significant (*p* < 2e-11) overexpression of five genes in clonal PCs with progression from MGUS to sMM and to overt MM (Figs. 8G, S4A). Kaplan–Meier survival curves showed that overexpression of *SNRPB* and *SLC25A5* are associated with significantly (*P* < 0.013, *P* < 0.012) shorter overall survival of MM patients, with a trend towards poor outcomes for *CLPP* and *MAPKAPK2* genes (Figs. 8H, S4B). SNRPB mediates spliceosome assembly, and SLC25A5 is a mitochondrial protein that regulates mitochondrial energy transfer [28–30]. The other two signature genes, *CLPP* and *UQCRFS*, encode a protease and a reductase, and are involved in mitochondrial metabolism. *MAPKAPK2*, *SNRPB*, and *SLC25A5* gene overexpression identified here are deregulated in MM and implicated in solid tumor progression [29, 46–48]. Based on our results, we speculate that the RAS mutation is not the only factor to predict treatment response to RAS inhibitors; in this process, MAPK pathway activation plays a critical role and should be considered [40].

## Discussion

Targeting the RAS-RAF-MEK-ERK and CDK pathway therapeutically in MM has long been a high priority. Extensive whole-genome sequencing analyses of >1000 MM patients confirm that RAS activating mutations and alterations in Cyclin-D pathways still remain as one of the most frequent recurrent genetic alterations detected in MM patients at the time of diagnosis or at relapse. Furthermore, RAS mutations distinguish MGUS from MM, indicating their crucial role in MM transformation [49]. Our group and others have previously demonstrated that small molecule inhibitors of MEK abrogate both constitutive and cytokine-stimulated p-ERK-mediated MM cell proliferation [7, 12–16, 35, 38, 39]. However, preclinical and clinical studies highlight the need to develop more selective and potent inhibitors targeting RAS/MEK pathway to improve MM patient outcome [12–14, 16, 35, 39, 50–52]. Here we tested the anti-MM activity of novel selective inhibitors targeting Erk1/2 and CDK4/6, which have been clinically evaluated in patients with metastatic solid tumors [20, 25–27].

Our data demonstrate that Erk1/2i therapy as a single agent may require different dosing strategies in patients, depending on their genetic and cytogenetic profiles. To enhance the efficacy of Erk1/2i on MM cell proliferation and overcome drug-induced resistance, we evaluated the combination Erk1/2i + CDK4/6i. CDK4 and CCDN1 or CDK4/6 and CCDN2 are overexpressed in MM; moreover, CDK6 overexpression (*CDKN2C* deletion) in MM correlates with poor overall survival [33, 53]. Importantly, our in vitro studies demonstrated highly synergistic in vitro MM cytotoxicity triggered by Erk1/2i + CDK4/6i treatment. Contrary, HD PBMC were relatively resistant to combined therapy, suggesting a favorable therapeutic index. The clinical potential of Erk1/2i + CDK4/6i was further supported by in vivo study demonstrating a significant *(P* = 0.0004) decrease of the MM burden in Erk1/2i + CDK4/6i-treated mice compared to the vehicle-treated control, without adverse effects.

To define the mechanism underlying the observed synergism between Erk1/2i and CDK4/6i, we evaluated their impact on MM cell cycle and apoptosis. Treatment of MM-cells with Erk1/2i and CDK4/6i, alone or in combination and in a dose-dependent manner, arrested MM-cells in G0/G1 phase. We observed a decrease in the expression/levels of Erk1/2, p-Erk1/2, and CDK4/6 in MM-cells treated with Erk1/2i + CDK4/6i compared to cells treated with either single agent. We also detected PARP cleavage and CAS3/7 activation in these MM-cells. Our studies, therefore, indicate ERK1/2i + CDK4/6i combination is more effective than single-agent treatment. Since Erk1/2 and CDK4/6 are key signaling molecules controlling MM cell proliferation and survival, targeting the combination may inhibit both pathways and thereby abrogate both MM cell growth and acquired drug resistance.

Activated Erk1/2 or CDK4/6 directly affects mitochondrial metabolism and potentiates the catalytic activity of some pro-apoptotic proteins [41, 42]. Therefore, we next assessed early death signaling in MM-cells after Erk1/2i + CDK4/6i treatment using dynamic BH3 profiling. Our study showed that cell treatment with Erk1/2i + CDK4/6i increased the priming response of H929, and MM1S cells to the BIM peptide, which targets all anti-apoptotic proteins (BCL2, BCL-XL, MCL1) in these cells [32]. Furthermore, MM-cells treated with ERK1/2i + CDK4/6i showed dose-dependent significant priming to peptides BAD and HRK, suggesting their dependency on BCL2 and BCL-XL anti-apoptotic proteins. Our studies showed priming dependency of MM1S cells on MCL-1 after Erk1/2i + CDK4/6 treatment. These results are consistent with prior reports that BCL2 and MCL1 proteins are overexpressed in a subset of MM patients and associated with shorter survival of these patients [54–56]. Our results indicate that the Erk1/2i + CDK4/6i combination activates mitochondrial apoptotic signaling in MM. These results are therefore consistent with the observed synergistic cytotoxicity of Erk1/2i and CDK4/6i combination treatment.

Our studies further evaluated the functional effects of Erk1/2i, CDK4/6i, and Erk1/2i + CDK4/6i, which demonstrated that combined treatment inhibits phospho-S6 and downregulates c-myc. Protein S6 is a part of the 40S ribosomal subunit and can be phosphorylated by S6 kinase in the mTOR pathway in an ERK-dependent manner [57, 58]. Our study shows that inhibition of phospho-S6 and downregulation of c-myc correlate with Erk1/2i + CDK4/6i-induced G0/G1 cell cycle arrest. Furthermore, treatment with Erk1/2i + CDK4/6i significantly decreased the levels of p-Rb and E2F1, downstream targets of CDK4/6, which is a master regulator of the cell cycle. Recent studies show that CDK4/6 also induces tumorigenesis through the regulation of inflammatory cytokines induced via (NF)-κB pathway activation [59, 60]. Therefore CDK4/6i may also modulate cytokines IL-6, TNF-a, VEGF which are known to mediate MM cell growth, survival and drug resistance in the BM-milieu. Our studies of MM patient samples indicate that MM cells co-cultured with or without autologous BMSC-CM remain equally sensitive to Erk1/2i + CDK4/6i, suggesting that this drug combination can overcome the protective effects of the MM-BM-milieu. Previous studies done by our group and others have shown MM BM milieu induced Erk1/2 activation in MM patients confers resistance to MM therapies including immunomodulatory drugs iMiDs [61, 62]. Indeed our most recent genome-wide CRISPR-Cas9 knockout screening identified and validated Erk1/2 as a CRBN-IKZF1/3 axis-independent modulator of BM-milieu-induced sensitivity to iMiDs; based on our current study, ERK1/2i + CDK4/6i may also overcome this mechanism of IMiD induced resistance in the tumor BM microenvironment [63].

We then went on to identify potential biomarkers predictive of responses to Erk1/2i + CDK4/6i in MM. Our study of global transcriptome alterations in response to Erk1/2i + CDK4/6i treatment in MM cells, with or without Erk1/2 knockdown, identified gene-sets that were commonly and persistently downregulated in cells treated with Erk1/2i + CDK4/6i. Validation studies conducted in two independent MM cohorts identified commonly deregulated genes associated with Erk1/2i + CDK4/6i treatment: *SNRPB, SLC25A5*, and *MAPKAPK2*. *SNRPB* is part of both pre-catalytic and activated spliceosome complexes. Overexpression of *SNRPB* leads to alterations in the splicing process in MM-cells of patients treated with proteasome inhibitors (PI) [46]. Recently, Wang et al identified a novel prognostic signature in MM based on expression of eight RBP’s (RNA binding protein) which includes SNRPB [64]. *MAPKAPK2* is known to be involved in transcriptome regulation via the activation/deactivation of RNA-binding proteins [65]. It is, therefore, possible that Erk1/2i + CDK4/6i treatment, in addition to inducing mitochondrial-dependent MM cell killing, may inhibit PI altered RNA splicing and overcome PI drug resistance in MM. Based on our studies and others, we suggest that the RAS/CDK axis might be involved in MM transformation and/or disease progression via alterations of splicing mechanism. The identified signature genes also include the *SLC25A5*, which facilitates ATP transfer in the inner mitochondrial membrane [30]. This suggests that Erk1/2i + CDK4/6i treatment may lead to suppression of MM cell metabolism.

Our studies demonstrate that Erk1/2i + CDK4/6i inhibited MM cell survival and induced mitochondrial-dependent apoptosis, associated with downregulation of key target molecules c-myc, p-RSK, p-S6, p-RB, and E2F1 in MM cell lines and patient cells with complex genetic profiles. Importantly, our studies showed synergistic anti-MM activity of Erk1/2i + CDK4/6i combination in vitro and in vivo, as well as identified biomarkers associated with response to this treatment. These data provide the preclinical framework for clinical trials evaluating combination Erk1/2i + CDK4/6i therapy to improve MM patient outcomes.

## Supplementary information

Supplementary Materials
