# Author Correction: Combination therapy targeting Erk1/2 and CDK4/6i in relapsed refractory multiple myeloma

**DOI:** 10.1038/s41375-022-01588-z

**Published:** 2022-05-05

**Authors:** Sophia Adamia, Shruti Bhatt, Kenneth Wen, Zuzana Chyra, Geoffrey G. Fell, Yu-Tzu Tai, Marissa S. Pioso, Ivane Abiatari, Anthony Letai, David M. Dorfman, Teru Hideshima, Kenneth C. Anderson

**Affiliations:** 1 Jerome Lipper Multiple Myeloma Disease Center, Dana-Farber Cancer Institute, Harvard Medical School, Boston, MA 02215 USA; 2 Dana-FArber Cancer Institute, Harvard Medical School, Boston, MA 02215 USA; 3 Department of Pharmacy, National University of Singapore, Singapore, 117559 Singapore; 4 Department of Data Science, Dana-Farber Cancer Institute, Boston, MA 02215 USA; 5 Ilia State University, School of Medicine, Tbilisi, G409 Georgia; 6 Department of Pathology, Brigham and Women’s Hospital, Harvard Medical School, Boston, MA 02215 USA

**Keywords:** Targeted therapies, Cancer

Correction to: *Leukemia* (2021) 36:1088–1101 10.1038/s41375-021-01475-z, published online 27 January 2022

The Acknowledgments section of this article was incomplete in the initial publication. it should read as follows:

Acknowledgments: We thank Catherine A. Nicholas for her help with preliminary experiments and Morgan F. O’Keefe for manuscript proofreading. This work was supported by the National Institutes of Health Specialized Programs of Research Excellence (SPORE) grant P50100707 and P01 CA155258; as well as by the Adelson Medical Research Foundation and the Paula and Rodger Riney Foundation.

