# Supplementary material for: Combination therapy targeting Erk1/2 and CDK4/6i in relapsed refractory multiple myeloma
Source: Leukemia. 2022 Jan 27;36(4):1088–101. doi: 10.1038/s41375-021-01475-z (PMC8979823; doi:10.1038/s41375-021-01475-z)
Supplement: Supplementary file 1 — Supplementary Materials [file 41375_2021_1475_MOESM1_ESM.pdf]

Supplementary Materials

Materials and Methods

S1 Western Blotting

The membranes were incubated with the following antibodies: Erk (#4695); p-Erk (#4377); c-myc (#5605); p-S6 (S235/236) (#4858); p-RSK (#11989); pRB (S807/811) (#8516); pRB (S780) (#8180); E2F1 (SC-251); CDK6 (#3136); p-CDK4(AP0593); PARP (#9542); p27 (#3686) and GAPDH (D16H-11) (#5174).

S2 Dynamic BH3 Profiling

MM cell lines were exposed to a panel of targeted agents or DMSO (control), followed by BH3 profiling using BH3 peptides, as previously described.[1] Briefly, cells were exposed to synthetic BH3 peptides after plasma membrane permeabilization with digitonin, and sensitivity to BH3 peptides was measured as cytochrome *c* loss via FACS, with DMSO-negative control depicting 100% cytochrome *c* retention and alamethecin used as a positive control for 100% cytochrome *c* release. The read-out for drug-induced change in priming is defined as “delta priming” (cytochrome *c* loss drug - cytochrome *c* loss DMSO).

S3 Orthotopic xenograft MM1S-luc MM model

Virus- and *Mycoplasma*-free MM1S-luc cells were washed, resuspended in 1X PBS, and administered to female NSG mice (6-8 weeks of age; The Jackson Laboratory, Bar Harbor, Maine) via IV tail vein injection (2 X 10^6 cells/250 ul PBS). LY3214996 and LY2835219 were formulated in 0.25% Tween80; mice (n=8) were treated 4 days after MM cell inoculation by oral gavage with vehicle qD (n=8) or LY3214996 (ERK1/2i, 100 mg/kg) and LY2835219 (CDK4/6i; 50 mg/kg) qD (n=8). Statistical significance of differences in bioluminescence measurements between the two study groups was determined using the Mann-Whitney test (two-tailed), and statistical significance for survival was

determined using both the Log-rank (Mantel-Cox) test and the Gehan-Breslow-Wilcoxon test. $P < 0.05$ was considered to be statistically significant.

S4 RNA seq and identification of treatment related molecular signature

Human Erk1/2 siRNA (siR-Erk1/2) and scrambled siRNA (SC) transfections were performed using the Neon transfection system (ThermoFisher). Transfection efficacy and Erk1/2 knockdown were evaluated at transcript and protein levels by RNA-seq analyses and western blotting, respectively. RNA seq data was analyzed using Partek Flow custom pipeline. RNA seq data were analyzed using the Partek Flow custom pipeline. Briefly, we identified significantly deregulated (up or downregulated) genes in H929 cells treated with Erk1/2i and CDK4/6i, alone or in combination, as compared to cells treated with DMSO. Using Venn methods, we identified genes selectively deregulated in Erk1/2i+CDK4/6i treated samples. We focused on genes that were downregulated (inhibited by Erk1/2i+CDK4/6i treatment) in this sample. We applied the same filtration pipeline to samples transfected with Erk1/2 siRNA and treated with Ei and Ci, alone or in combination. Next, using the Venn method, we were able to identify commonly deregulated genes between samples with and without Erk1/2 knockdown by siRNA and treated with Erk1/2i+CDK4/6i. At each filtration step, we selected significantly deregulated genes (p=0.005).

Target gene annotation followed by the gene and pathway enrichment analyses were done using the Database for Annotation, Visualization, and Integrated Discovery (DAVID), the Cancer Gene Census (CGC), and The Drug Gene Interaction Database (DGIdb). For validation studies, target gene expression profiles were evaluated in two MM cohorts. One cohort includes 41-MM, 33 monoclonal gammopathy of undetermined significance (MGUS), 33 smoldering MM (sMM), and 5-HD samples (GDS4968); while the second cohort includes 559 MM, 44 MGUS, 12 sMM, and 22 HD (GSE5900, 2658) CD138+ BM samples. Data were analyzed using the Partek Genomics Suite, according to the standard pipeline.

1. Montero J, Sarosiek KA, DeAngelo JD, Maertens O, Ryan J, Ercan D, Piao H, Horowitz NS, Berkowitz RS, Matulonis U, Janne PA, Amrein PC, Cichowski K, Drapkin R, Letai A. Drug-induced death signaling strategy rapidly predicts cancer response to chemotherapy. *Cell*. 2015;160(5):977-989.

Fig. S1.

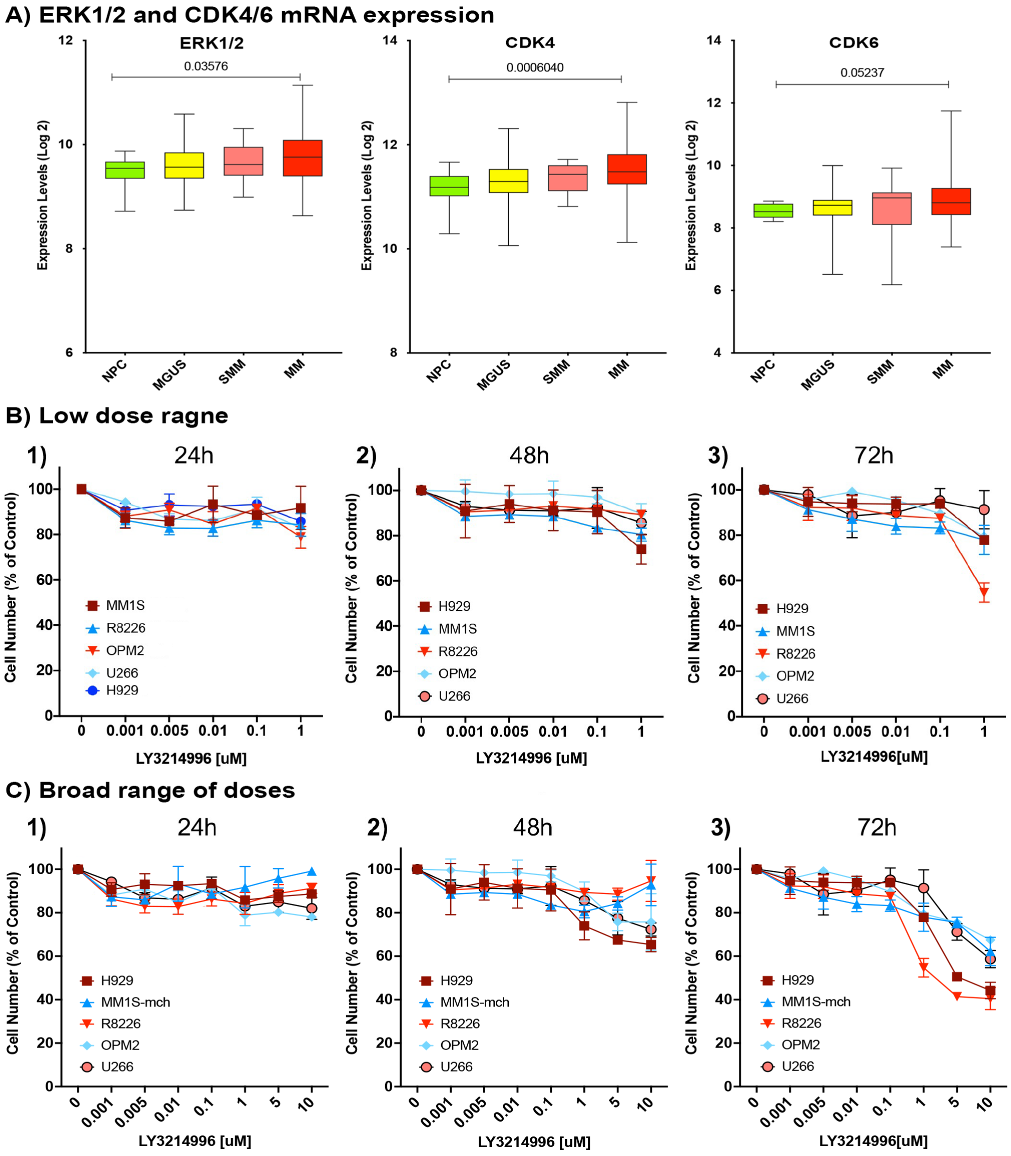

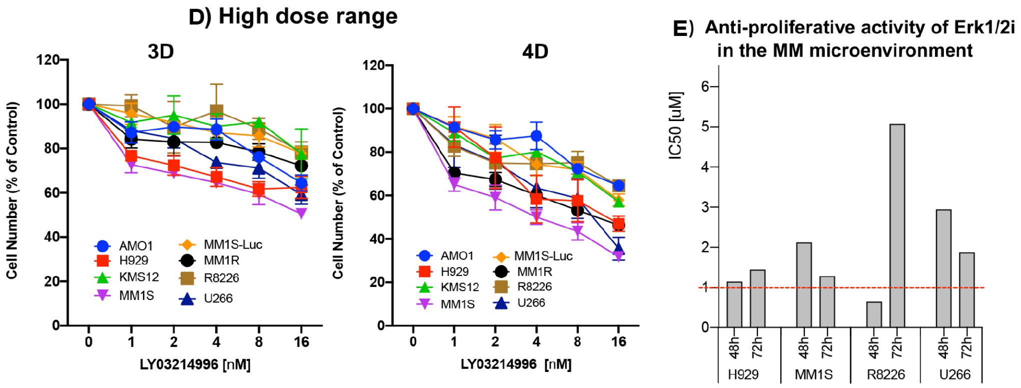

Fig. S1. Effects of LY3214996 (Erk1/2) in MM cell lines and healthy donors (HDs). Box plots showing *ERK1/2* and *CDK4/6* gene expression values in CD138+ PC from monoclonal gammopathy of undetermined significance (MGUS), multiple myeloma (MM), smoldering MM (sMM), and NPC (healthy donors), from dataset GSE2658-GSE5900. The error bars show the standard deviation. Horizontal bars indicate the mean value. The x-axis shows the samples analyzed, and the y-axis displays the expressions at log2 fold. These analyses confirmed significant *(0.05 >P< 0.0006)* overexpression of *ERK1/2, CDK4 and CDK6* transcripts in PCs. **(A);** RAS mutated (MM1S, H929, R8226) and wild type (WT) (OPM2, U266) cell lines were treated for 24h, 48h, 72h (3D), or 4 days (4D) with DMSO or Erk1/2i (LY3214996, 0-1uM) in RPMI medium with 0.1% DMSO and 10%FBS. Cells were treated with LY3214996 (0; 0.001; 0.005; 0.1; 1uM (**B**); 0.001; 0.005; 0.01; 0.1; 1; 5; 10uM **(C); or** 0; 1; 2; 4; 8; 16 uM **(D).** Growth was assayed by CellTiter-Glo assay. **(E)** Anti-proliferative activity of ERK1/2i in the MM microenvironment. BMSC-conditioned media (BMSC-CM) was collected from BMSCs cultured for three days in RPMI medium supplemented with 5% FBS. BMSC-CM was filtered with 0.2-micron syringe filter before use. On the Figure IC50s are shown for H929, MM1S, R8266, and U266 cell lines treated with DMSO or Erk1/2i (LY3214996, 0–16 uM) with or without BMSC-CM for 48 h and 72 h. IC50 was determined by non-linear regression using Prism, version 8.

Fig. S2.

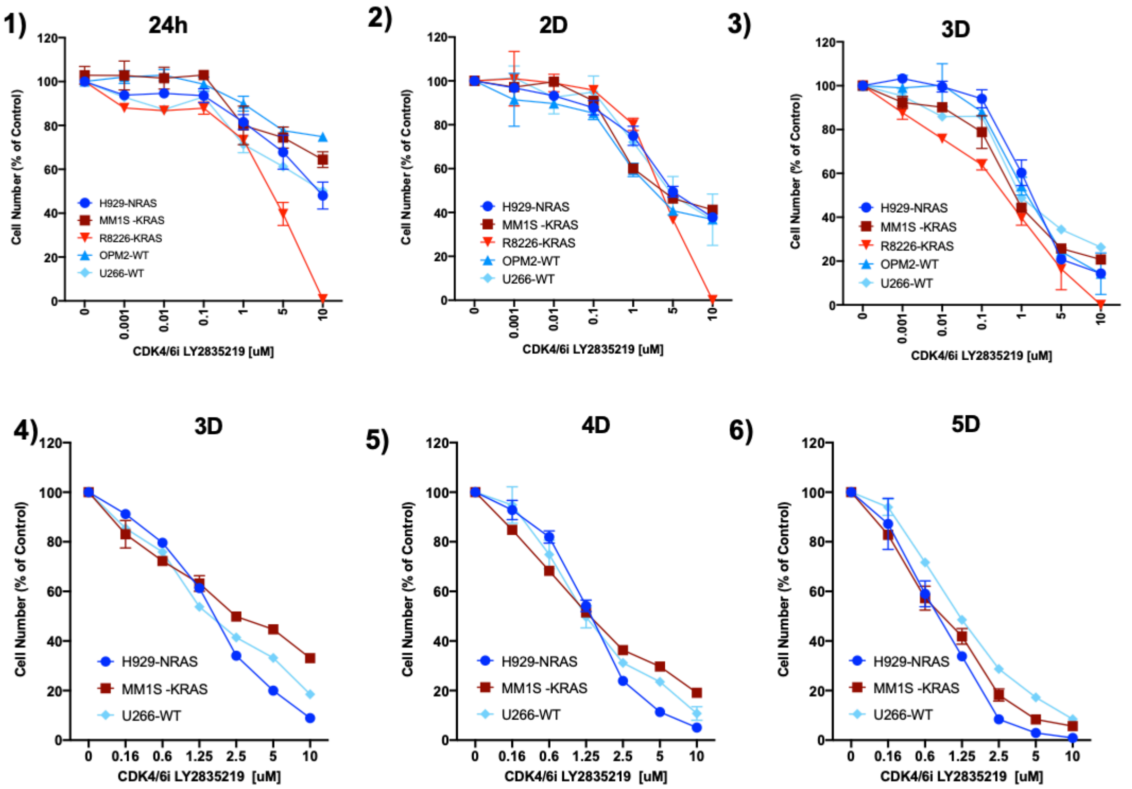

Fig. S2. Effects of LY2835219 (CDK4/6i) in MM cell lines and HDs. RAS mutated (MM1S, H929, R8226) and wild type (WT) (OPM2, U266) cell lines were treated for 24h, 48h (2D), 72h (3D), 4D and 5D with DMSO or LY2835219 (0; 0.001; 0.005; 0.1; 1uM or 0; 0.16; 0.6; 1.25; 2.5; 5; 10uM) in RPMI medium with 0.1% DMSO and 10%FBS.

Fig. S3

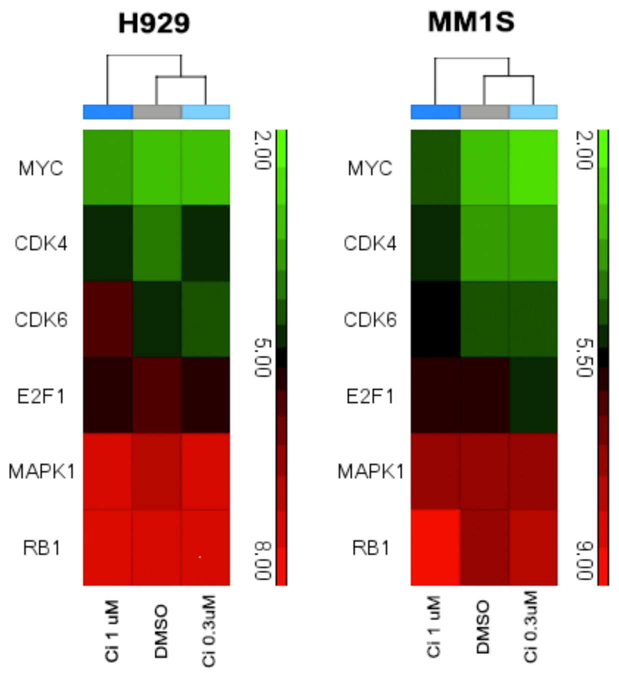

Fig. S3. Total mRNAs were isolated from MM1S and H929 cells and transcribed into cDNAs. Gene expression profiling was performed using custom TaqMan assays. Results were analyzed using the relative standard curve method, and final data analyses and heatmaps were generated based on dCt values using Partek Genomic Suite. Expression levels (dCts) are indicated with intensity shades of green/red colors. GAPDH and TBP genes were used as housekeeping genes.

Fig. S4.

A)

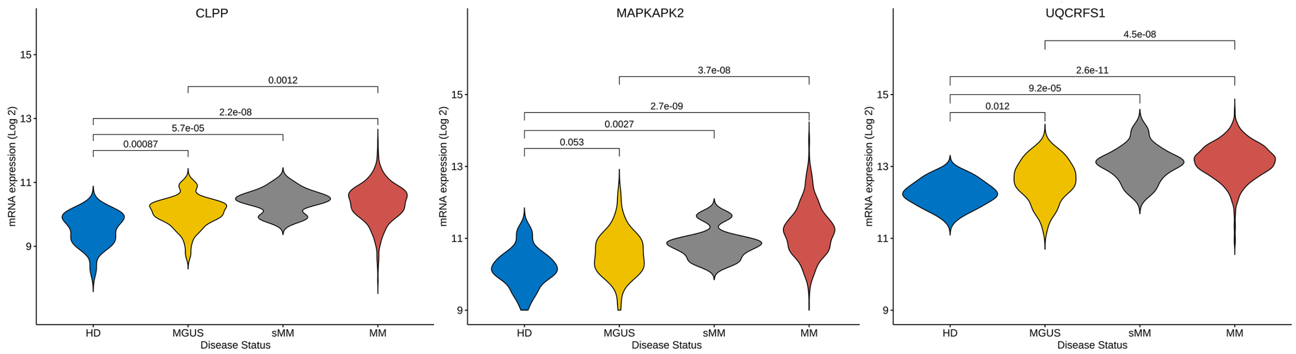
CLPP
MAPKAPK2
UQCRFS1
mRNA expression (Log 2)
HD
MGUS
sMM
MM
Disease Status
0.00087
5.7e-05
2.2e-08
0.0012
0.053
0.0027
2.7e-09
3.7e-08
0.012
9.2e-05
2.6e-11
4.5e-08

B)

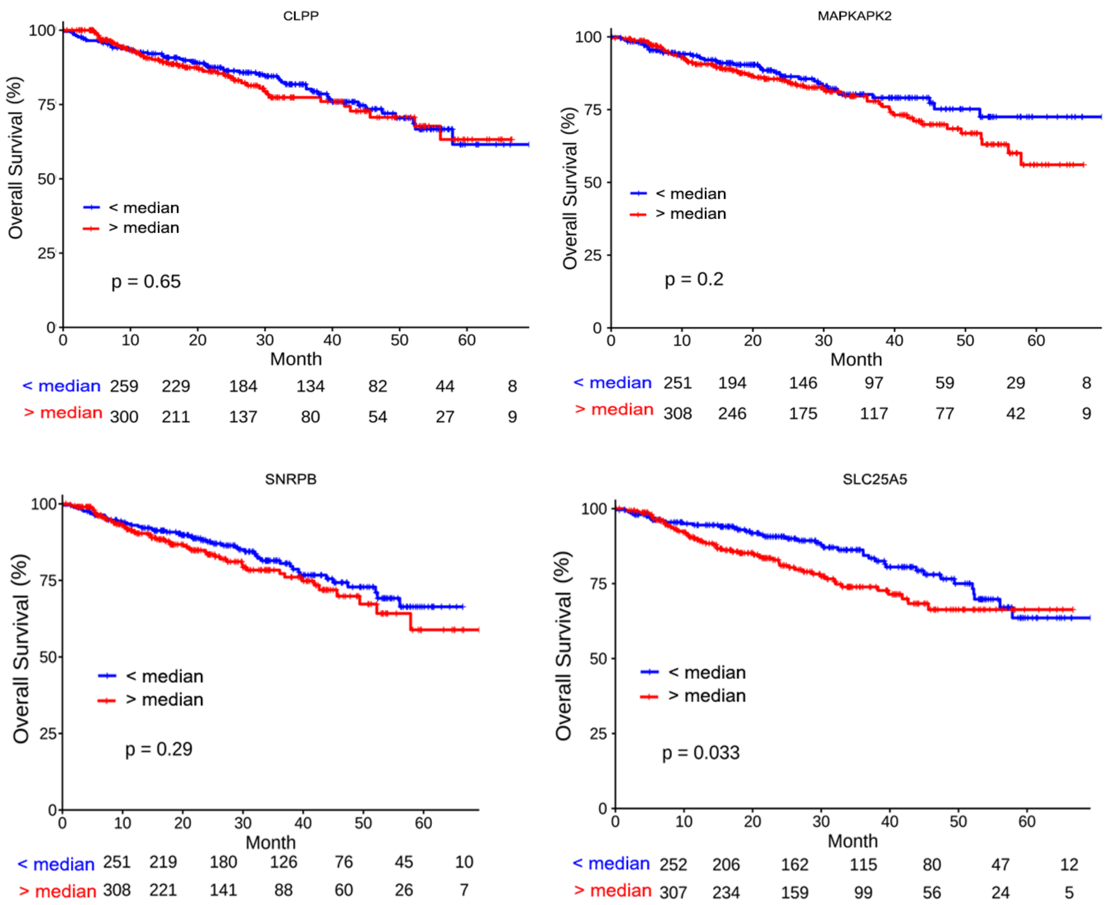
CLPP
MAPKAPK2
SNRPB
SLC25A5
Overall Survival (%)
Month
< median
> median
p = 0.65
p = 0.2
p = 0.29
p = 0.033
< median 259 229 184 134 82 44 8
> median 300 211 137 80 54 27 9
< median 251 194 146 97 59 29 8
> median 308 246 175 117 77 42 9
< median 251 219 180 126 76 45 10
> median 308 221 141 88 60 26 7
< median 252 206 162 115 80 47 12
> median 307 234 159 99 56 24 5

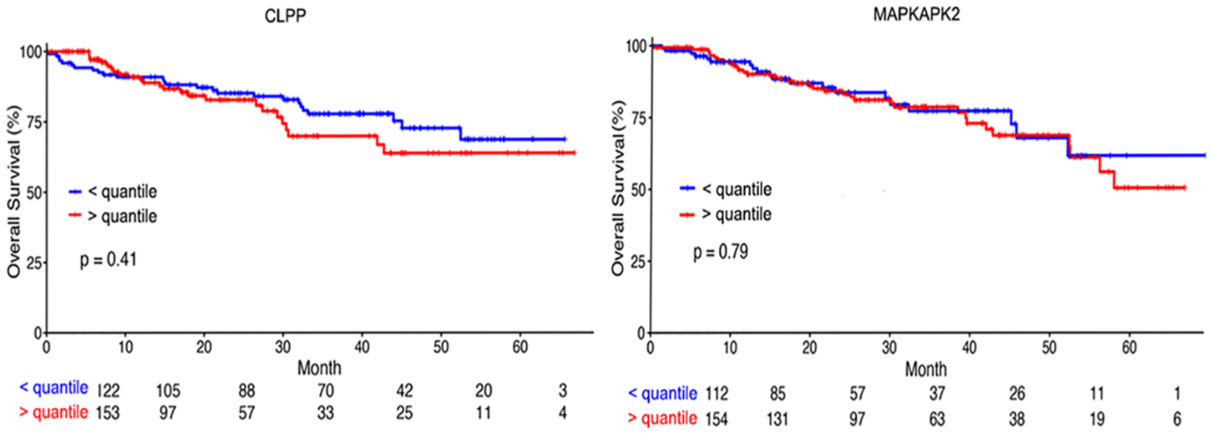

Fig. S4. Overexpression of Erk1/2i+CDK4/6i signature genes in MM patients and correlation analyses (A) Violin plots showing gene expression values in CD138+ PC from MGUS (n=44), sMM (n=12), MM (n=559) and HD (healthy donor, n=22), from datasets GSE5900-GSE2658. The x-axis shows the samples analyzed; violin plots are colored by sample type. The y-axis displays the expression at log2 fold. Significance between groups was evaluated using a Wilcoxon Rank-Sum test; the type I error cut off was 5%. Multiple testing adjustments between statuses were then made using the Bonferroni correction. These analyses show significant overexpression of *CLPP, MAPKAPK2 and UQCRF1,* and *SLC25A5* transcripts in PCs from all patient groups analyzed.

(B) The relevance of *CLPP, MAPKAPK2, SLC25A5, SNRPB and SLC25A5* expression to clinical outcomes was estimated in 559 MM patient samples. The samples were classified based on expression levels. Survival curves were estimated using the Kaplan-Meier method; the type I error cut off was 5%. Differences in survival were assessed using the log-rank test and Cox regression models. For analyses R version 4.0.0, along with the survival and survminer packages, were used. Samples were classified based on median (> or <) or quantile (<25% or >75%) expressions.

Fig. S5.

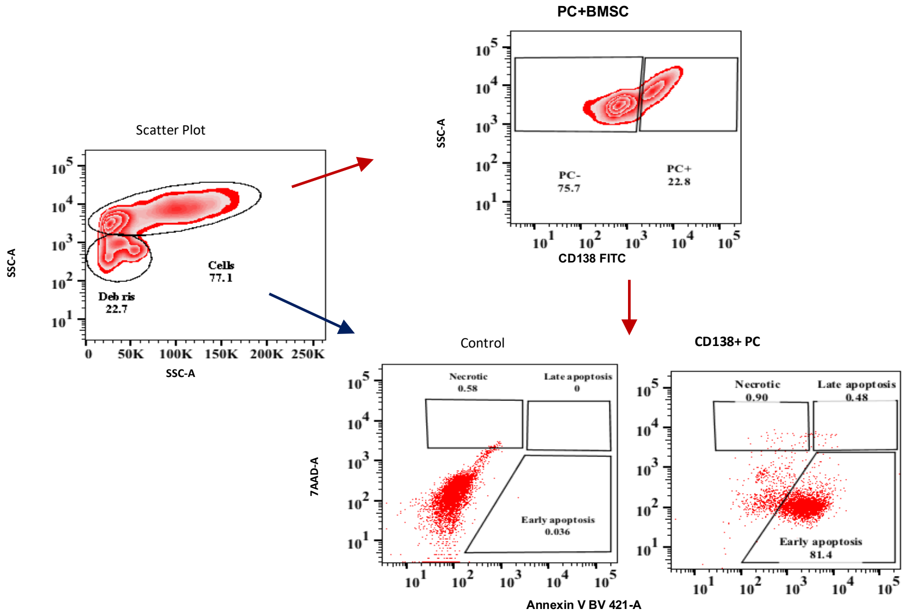

Fig. S5. Gating strategy – representative flow cytometry data to illustrate gating strategy for apoptosis assay. Unfractionated BM cells from MM patients were treated with Erk1/2i and CDK4/6i, alone and combination. Cells were harvested 3D after treatment and stained with anti CD138 antibodies 15 min prior to annexin V and 7-AAD staining. Apoptotic cells measured on the BD Fortessa X-20, followed by analysis using FlowJo software.

Table S1A G-set 1. Upregulated genes in Erk1/2 knockdown cells

Gene symbol	Chromosome	P-value	Fold change	FDR
RAVER1	19	0.00118	5.69	0.0338
ANKLE1	19	0.00041	3.70	0.0144
AMPD1	1	0.00000	3.32	0.0000
RAB35	12	0.00026	3.26	0.0098
TNFRSF17	16	0.00000	3.14	0.0000
SLC39A4	8	0.00105	3.05	0.0307
LIMD1	3	0.00000	3.02	0.0002
MCM4	8	0.00010	2.94	0.0046
EIF3CL	16	0.00002	2.88	0.0010
CASP8	2	0.00067	2.78	0.0213
THAP7	22	0.00132	2.73	0.0364
TM9SF1	14	0.00099	2.66	0.0292
CTPS1	1	0.00035	2.64	0.0128
GABRR2	6	0.00003	2.64	0.0017
FH	1	0.00035	2.64	0.0128
MPV17	2	0.00030	2.59	0.0115
SEC61B	9	0.00000	2.58	0.0000
UQCRFS1	19	0.00002	2.55	0.0012
FOXRED2	22	0.00032	2.49	0.0121
AKAP13	15	0.00138	2.49	0.0375
NDUFB8	10	0.00001	2.47	0.0004
TXLNA	1	0.00048	2.44	0.0163
PRRC2B	9	0.00117	2.43	0.0336
CLPP	19	0.00062	2.40	0.0202
ARID1A	1	0.00007	2.40	0.0035
IFNAR2	21	0.00023	2.40	0.0089
GNL3L	X	0.00037	2.39	0.0134
PRDM15	21	0.00002	2.37	0.0009
PKD1	16	0.00004	2.36	0.0019
IRF4	6	0.00000	2.36	0.0000
PPP1R10	6	0.00012	2.35	0.0054
TRUB2	9	0.00069	2.33	0.0218
CHPF	2	0.00000	2.31	0.0000
ZNF512B	20	0.00008	2.30	0.0038
POP7	7	0.00124	2.29	0.0347
ATP6V0C	16	0.00001	2.29	0.0004
TRIM65	17	0.00060	2.29	0.0198
NDUFV3	21	0.00035	2.28	0.0128
PCGF3	4	0.00121	2.27	0.0341
SPCS2	11	0.00005	2.25	0.0025
MRI1	19	0.00042	2.25	0.0146
GRWD1	19	0.00001	2.22	0.0006

ISG20	15	0.00001	2.22	0.0006
GATD1	11	0.00000	2.22	0.0001
HSPA4	5	0.00001	2.21	0.0006
EDEM3	1	0.00000	2.20	0.0000
POLR2K	8	0.00116	2.20	0.0335
SLC25A5	X	0.00000	2.18	0.0000

Table S1B G-set 1. Cancer related genes

Gene symbol	Chromosome	P-value	Fold change	FDR
AMPD1	1	0.00000	3.32	0.0000
ATIC	2	0.00049	2.13	0.0166
CASP8	2	0.00067	2.78	0.0213
FGFR3	4	0.00000	2.01	0.0000
FH	1	0.00035	2.64	0.0128
ID3	1	0.00122	2.04	0.0343
IRF4	6	0.00000	2.36	0.0000
PRDM1	6	0.00001	2.18	0.0008
TNFRSF17	16	0.00000	3.14	0.0000

Table S1C G-set-1. Gene and Pathway enrichment analyses

Biloical function	P Value	Fold Enrichment
JAK-STAT cascade	4.3E-04	26.6
mRNA splicing, via spliceosome	3.7E-03	5.7
FGFR	6.6E-03	10.4
B cell activation	8.0E-03	22.0
transcription, DNA-templated	1.7E-02	1.8
proteolysis	2.1E-02	13.3
mitochondrial electron transport, NADH to ubiquinone	2.2E-02	13.0
de novo' IMP biosynthetic process	2.8E-02	70.9
cell proliferation	2.8E-02	3.5
ER-associated ubiquitin-dependent protein catabolic process	3.2E-02	10.6
mitochondrial respiratory chain complex I assembly	3.5E-02	10.1
type I interferon signaling pathway	3.6E-02	10.0
endosomal transport	3.8E-02	9.7
chromatin-mediated maintenance of transcription	4.1E-02	47.2
placenta blood vessel development	4.6E-02	42.5
transcription-coupled nucleotide-excision repair	4.6E-02	8.6

Table S2A G-set 2. Downregulated genes in response to Erk1/2i+CDK4/6i treatment

Gene symbol	Chromosome	P-value	Fold change	FDR
TYMS	18	2.6E-05	-86.84	2.69E-04
RRM2	2	7.5E-15	-59.64	2.85E-13
DUSP6	12	9.8E-12	-53.55	2.81E-10
PCLAF	15	3.2E-09	-51.33	6.73E-08
CDK1	10	3.7E-05	-50.85	3.65E-04
STMN1	1	6.2E-15	-40.55	2.34E-13
FAM111B	11	6.6E-04	-38.65	4.51E-03
NUF2	1	1.0E-09	-37.25	2.30E-08
MYEOV	11	3.0E-09	-33.76	6.34E-08
BIRC5	17	2.7E-08	-28.31	4.91E-07
PKMYT1	16	6.7E-06	-27.89	7.76E-05
MKI67	10	4.4E-13	-26.97	1.45E-11
NEK2	1	3.9E-09	-26.77	7.98E-08
HMMR	5	1.6E-04	-26.49	1.34E-03
TOP2A	17	1.6E-13	-26.36	5.38E-12
AURKB	17	1.9E-07	-22.71	3.02E-06
CCNB2	15	1.9E-05	-21.01	1.96E-04
CDCA2	8	3.1E-04	-20.97	2.35E-03
GINS2	16	1.2E-05	-20.33	1.37E-04
SKA1	18	2.1E-06	-20.26	2.66E-05
KIF20A	5	1.1E-09	-19.20	2.47E-08
NCAPG	4	1.1E-09	-19.11	2.37E-08
DTL	1	6.5E-05	-18.74	6.02E-04
HJURP	2	1.7E-09	-18.42	3.63E-08
ASPM	1	3.4E-06	-18.15	4.21E-05
CDKN3	14	3.0E-05	-18.15	3.01E-04
CDC25A	3	4.8E-04	-17.88	3.40E-03
WDR76	15	1.2E-03	-17.83	7.50E-03
CDT1	16	4.0E-14	-17.76	1.44E-12
TPX2	20	1.7E-12	-17.43	5.20E-11
CENPF	1	1.5E-11	-16.53	4.23E-10
CDCA5	11	4.3E-07	-16.29	6.30E-06
TMSB4X	X	1.3E-16	-16.24	5.71E-15
CENPM	22	8.2E-05	-15.96	7.31E-04
CDC45	22	8.9E-04	-15.72	5.81E-03
UBALD2	17	2.7E-20	-15.66	1.45E-18
HMGB2	4	1.3E-37	-15.41	1.43E-35
SHCBP1	16	1.7E-10	-15.34	4.26E-09
PRR11	17	8.6E-08	-15.13	1.44E-06
TK1	17	1.3E-12	-14.44	4.04E-11
CCNA2	4	4.3E-08	-14.14	7.63E-07
UHRF1	19	1.6E-06	-13.99	2.13E-05

LMNB1	5	3.1E-12	-13.85	9.16E-11
KIFC1	6	9.4E-07	-13.75	1.31E-05
ESPL1	12	8.9E-05	-13.34	7.87E-04
SPC24	19	7.2E-08	-13.14	1.23E-06
CCNB1	5	6.1E-17	-12.48	2.69E-15
MAD2L1	4	1.9E-04	-12.10	1.56E-03
TROAP	12	2.3E-10	-11.97	5.68E-09
UBE2C	20	1.6E-16	-11.89	6.83E-15
PLK1	16	8.3E-12	-11.82	2.39E-10
BGLAP	1	1.1E-18	-11.66	5.22E-17
PCNA	20	1.2E-11	-11.25	3.49E-10
KIF11	10	1.4E-06	-11.24	1.86E-05
GGH	8	2.1E-07	-11.18	3.31E-06
MB	22	1.3E-06	-11.16	1.73E-05
AURKA	20	6.3E-10	-11.08	1.46E-08
TUBA1B	12	3.9E-88	-11.06	1.22E-85
SAP30	4	1.1E-04	-10.91	9.31E-04
ASF1B	19	7.4E-06	-10.76	8.45E-05
UNG	12	2.7E-09	-10.70	5.61E-08
NCAPH	2	1.6E-04	-10.70	1.34E-03
ATAD2	8	4.2E-09	-10.64	8.66E-08
SMC2	9	3.3E-05	-10.59	3.26E-04
MCM5	22	8.2E-21	-10.54	4.68E-19
STS	X	1.4E-05	-10.47	1.49E-04
PRC1	15	7.3E-07	-10.43	1.05E-05
PTTG1	5	6.1E-09	-10.40	1.22E-07
CHEK1	11	2.6E-07	-10.17	3.94E-06
CDC20	1	1.1E-12	-10.10	3.44E-11
CERKL	2	3.0E-10	-9.98	7.38E-09
NEMP1	12	1.4E-03	-9.92	8.68E-03
CENPU	4	1.1E-04	-9.78	9.16E-04
DEK	6	5.7E-06	-9.46	6.72E-05
BUB1	2	8.5E-12	-9.40	2.45E-10
NDC80	18	9.7E-11	-9.34	2.50E-09
CCL3	17	4.8E-15	-9.30	1.86E-13
DHFR	5	6.4E-13	-9.23	2.06E-11
CKS1B	1	1.7E-07	-9.22	2.67E-06
ASB2	14	5.9E-16	-9.21	2.42E-14
MCM6	2	2.1E-10	-9.06	5.17E-09
C1orf112	1	1.1E-03	-9.03	7.21E-03
APOBEC3B	22	1.4E-14	-8.98	5.27E-13
ZWINT	10	2.3E-05	-8.97	2.34E-04
SPAG5	17	1.1E-08	-8.96	2.14E-07
CDCA8	1	3.1E-04	-8.91	2.36E-03
CEP55	10	6.7E-06	-8.89	7.78E-05

NUSAP1	15	2.7E-11	-8.88	7.51E-10
KIF2C	1	7.7E-06	-8.75	8.75E-05
MSH2	2	7.2E-08	-8.70	1.22E-06
MCM4	8	1.8E-48	-8.69	2.52E-46
TTK	6	8.1E-05	-8.62	7.23E-04
CHAF1A	19	3.1E-10	-8.54	7.44E-09
DEPDC1	1	1.1E-04	-8.48	9.76E-04
F2R	5	6.7E-17	-8.46	2.98E-15
MYBL2	20	6.6E-20	-8.43	3.49E-18
CENPH	5	8.7E-06	-8.38	9.82E-05
C12orf75	12	1.4E-05	-8.22	1.55E-04
FOXM1	12	6.8E-05	-8.18	6.22E-04
KPNA2	17	1.0E-19	-8.00	5.14E-18
RRM1	11	9.8E-12	-7.89	2.81E-10
FCMR	1	1.4E-03	-7.61	8.73E-03
CENPE	4	6.8E-04	-7.47	4.64E-03
ARHGAP11A	15	3.6E-05	-7.34	3.56E-04
MTDH	8	3.9E-141	-7.25	1.98E-138
UBASH3B	11	4.1E-05	-7.20	3.94E-04
S100A4	1	6.1E-13	-7.17	1.98E-11
FEN1	11	1.9E-15	-7.16	7.58E-14
CCND1	11	3.2E-08	-7.13	5.84E-07
YWHAH	22	1.9E-06	-7.06	2.43E-05
HAT1	2	1.7E-05	-6.92	1.83E-04
PRIM1	12	5.6E-05	-6.84	5.23E-04
PAQR4	16	2.0E-04	-6.83	1.63E-03
BUB1B	15	2.7E-05	-6.79	2.70E-04
CENPL	1	1.1E-03	-6.70	6.97E-03
IRF2	4	4.6E-08	-6.69	8.10E-07
WEE1	11	1.6E-04	-6.60	1.30E-03
C1orf21	1	2.3E-04	-6.58	1.83E-03
TUBA1C	12	2.0E-19	-6.56	1.00E-17
TMPO	12	1.7E-20	-6.56	9.53E-19
SAPCD2	9	9.4E-08	-6.52	1.59E-06
RTCB	22	1.2E-08	-6.49	2.33E-07
CAP1	1	5.9E-12	-6.40	1.73E-10
TACC3	4	1.1E-09	-6.40	2.50E-08
SLBP	4	1.1E-09	-6.38	2.36E-08
GMNN	6	3.8E-04	-6.38	2.78E-03
CD68	17	2.0E-04	-6.27	1.59E-03
MCM2	3	5.7E-15	-6.21	2.17E-13
PTP4A1	6	3.5E-20	-6.12	1.87E-18
NCAPD2	12	2.9E-05	-6.12	2.94E-04
SUV39H1	X	7.1E-04	-6.12	4.81E-03
P4HA1	10	2.4E-17	-6.08	1.09E-15

Table S2B G-set 2. Cancer related genes

Gene symbol	Chromosome	P-value	Fold change	FDR
RPL10	X	6.2E-104	-2.39	2.58E-101
PABPC1	8	1.8E-69	-2.94	3.93E-67
HSP90AB1	6	1.3E-64	-2.04	2.72E-62
HSP90AA1	14	6.5E-56	-4.94	1.11E-53
PCBP1	2	5.9E-48	-3.92	8.22E-46
TNFRSF17	16	1.3E-42	-2.23	1.54E-40
HNRNPA2B1	7	2.4E-41	-2.24	2.87E-39
TPM3	1	2.8E-31	-2.72	2.50E-29
RPL5	1	3.1E-29	-2.00	2.59E-27
HMGA1	6	2.0E-27	-2.55	1.58E-25
HOOK3	8	4.4E-27	-3.26	3.32E-25
PTPN6	12	6.6E-26	-4.16	4.84E-24
SRSF3	6	6.0E-25	-3.75	4.07E-23
CNBP	3	1.4E-21	-2.03	8.59E-20
RAD21	8	1.3E-20	-4.78	7.13E-19
TOP1	20	1.3E-20	-3.41	7.15E-19
SET	9	9.0E-20	-3.29	4.67E-18
SFPQ	1	4.5E-19	-2.52	2.25E-17
MSN	X	1.9E-16	-3.65	8.18E-15
ID3	1	1.3E-15	-3.19	5.20E-14
FEN1	11	1.9E-15	-7.16	7.58E-14
RPN1	3	1.0E-14	-2.04	3.93E-13
YWHAE	17	1.3E-14	-2.88	4.81E-13
APOBEC3B	22	1.4E-14	-8.98	5.27E-13
IDH2	15	3.8E-14	-2.97	1.39E-12
NONO	X	4.1E-14	-2.14	1.47E-12
RHOA	3	2.2E-13	-2.48	7.34E-12
EZR	6	6.1E-13	-3.50	1.98E-11
SDHD	11	1.4E-12	-2.37	4.38E-11
CDKN1A	6	3.9E-12	-3.72	1.16E-10
TPM4	19	4.0E-11	-4.53	1.08E-09
CD28	2	5.7E-11	-2.21	1.50E-09
ATP1A1	1	1.6E-09	-3.05	3.52E-08
CLTC	17	1.8E-09	-2.62	3.95E-08
DDX3X	X	4.9E-09	-2.36	1.01E-07
SSX1	X	5.0E-09	-3.07	1.02E-07
CDK6	7	1.0E-08	-2.92	1.96E-07
NRAS	1	1.3E-08	-2.15	2.44E-07
CCND1	11	3.2E-08	-7.13	5.84E-07
MSH2	2	7.2E-08	-8.70	1.22E-06
STAG2	X	8.5E-08	-2.37	1.44E-06
LASP1	17	2.1E-07	-3.04	3.28E-06

CBFB	16	2.2E-07	-4.28	3.38E-06
EIF1AX	X	2.4E-07	-2.81	3.77E-06
FLI1	11	2.5E-07	-3.27	3.84E-06
NDRG1	8	3.9E-07	-3.03	5.74E-06
DDX10	11	7.1E-07	-3.30	1.02E-05
CASP3	4	2.5E-06	-2.82	3.17E-05
DEK	6	5.7E-06	-9.46	6.72E-05
GMPS	3	7.4E-06	-3.90	8.45E-05
CREB3L2	7	8.6E-06	-2.46	9.71E-05
SDHC	1	1.9E-05	-2.69	1.99E-04
MSH6	2	2.0E-05	-3.04	2.04E-04
BUB1B	15	2.7E-05	-6.79	2.70E-04
TCEA1	8	4.1E-05	-2.70	3.98E-04
MAP2K1	15	6.3E-05	-2.77	5.82E-04
SDHB	1	9.6E-05	-2.83	8.34E-04
SMC1A	X	9.6E-05	-3.17	8.40E-04
RFWD3	16	1.1E-04	-2.74	9.50E-04

Table S2C G-set-2. Gene and Pathway enrichment analyses

**Biloical function**	**P Value**	**Fold Enrichment**	**FDR**
Cell cycle	7.5E-24	5.6	1.8E-21
Oocyte meiosis	1.8E-10	3.9	2.2E-08
Spliceosome	9.5E-10	3.5	7.5E-08
DNA replication	4.4E-09	6.4	2.6E-07
Biosynthesis of antibiotics	3.2E-08	2.7	1.5E-06
Ribosome	1.1E-07	3.1	4.4E-06
RNA transport	1.7E-07	2.8	5.6E-06
Carbon metabolism	5.0E-07	3.2	1.5E-05
Glycolysis / Gluconeogenesis	1.8E-06	3.9	4.7E-05
Viral carcinogenesis	2.4E-05	2.3	5.5E-04
Proteasome	2.8E-05	4.3	5.5E-04
Pathogenic Escherichia coli infection	2.8E-05	4.0	5.5E-04
p53 signaling pathway	5.4E-04	3.0	9.9E-03
Mismatch repair	6.6E-04	5.0	1.1E-02
Biosynthesis of amino acids	1.1E-03	2.8	1.7E-02
Pyruvate metabolism	1.3E-03	3.6	1.9E-02
Bacterial invasion of epithelial cells	2.4E-03	2.6	3.3E-02
Citrate cycle (TCA cycle)	3.6E-03	3.9	4.7E-02
Shigellosis	3.9E-03	2.7	4.9E-02
Progesterone-mediated oocyte matur	6.2E-03	2.3	7.0E-02
Base excision repair	6.3E-03	3.5	7.0E-02
mRNA surveillance pathway	9.1E-03	2.2	9.7E-02
Regulation of actin cytoskeleton	9.5E-03	1.7	9.7E-02

Table S3 G-set-1- Cohort 1 MM vs. HD

Gene symbol	P-value	FDR	Fold change (log 2)
NDUFB8	4.9E-11	3.8E-09	2.0
SEC61B	2.7E-09	1.0E-07	2.2
SNRPB	6.3E-08	1.2E-06	2.1
NDUFA6	1.5E-07	2.3E-06	2.1
SLC25A5	4.5E-06	5.0E-05	2.1
CLPP	7.3E-05	6.4E-04	1.5
POP7	8.4E-05	6.6E-04	1.5
UQCRFS1	1.1E-03	5.3E-03	1.6
UBL5	4.6E-03	2.1E-02	1.4
ZNF593	6.4E-03	2.6E-02	1.4
GABRR2	1.6E-02	5.4E-02	1.3
CTPS1	1.6E-02	5.4E-02	1.5
SLC2A11	1.7E-02	5.4E-02	1.2
UBB	2.6E-02	7.8E-02	1.3
SLC19A1	3.0E-02	8.6E-02	1.2
TRUB2	3.5E-02	9.3E-02	1.5
MAPKAPK2	4.0E-02	1.0E-01	1.6
TOMM6	4.2E-02	1.0E-01	1.3
NUDC	5.3E-02	1.2E-01	1.2

Gene symbol	P-value	FDR	Fold change (log 2)
CHST15	1.3E-08	3.4E-07	-2.3
LIMD1	2.2E-06	2.9E-05	-2.3
AMPD1	3.9E-05	3.8E-04	-4.2
EDEM3	1.4E-04	1.0E-03	-2.7
PIAS1	2.1E-04	1.3E-03	-1.8
ARID1A	2.2E-04	1.3E-03	-1.7
PPP1R10	4.0E-04	2.2E-03	-1.8
IFNAR2	8.2E-04	4.3E-03	-1.8
PRRC2B	6.4E-03	2.6E-02	-1.4
TXLNA	9.3E-03	3.6E-02	-1.3
PCGF3	1.5E-02	5.4E-02	-1.3
BTAF1	1.7E-02	5.4E-02	-1.6
MPV17	3.1E-02	8.6E-02	-1.4
ZNF740	3.6E-02	9.3E-02	-1.2

G-set-1- Cohort 2 MM vs. HD

Gene symbol	P-value	FDR	Fold change (log 2)
POP7	2.8E-19	1.1E-17	2.9
SLC39A4	7.1E-09	2.2E-08	2.6
RAB35	2.2E-19	1.1E-17	2.4
ZNF593	2.2E-11	9.2E-11	2.3
CTPS1	5.3E-13	3.7E-12	2.2
MAPKAPK2	1.1E-11	5.8E-11	2.1
SLC25A5	2.0E-11	8.9E-11	2.0
ATIC	5.4E-19	1.4E-17	2.0
FH	5.2E-10	2.0E-09	2.0
MRPS16	3.3E-17	5.0E-16	1.9
NDUFB8	8.9E-19	1.7E-17	1.9
UQCRFS1	2.3E-15	2.5E-14	1.9

PAICS	3.8E-16	4.8E-15	1.9
SNRPB	9.3E-10	3.4E-09	1.9
PRRC2B	6.2E-09	2.0E-08	1.9
CLPP	9.3E-13	5.9E-12	1.8
UBL5	5.3E-09	1.7E-08	1.8
PPP1R10	1.3E-12	7.3E-12	1.8
SSBP1	1.4E-11	6.9E-11	1.7
PA2G4	1.0E-13	8.5E-13	1.7
MPV17	5.5E-05	1.2E-04	1.7
PSMB3	4.1E-09	1.4E-08	1.6
MCM4	2.3E-03	4.1E-03	1.6
NDUFA6	6.6E-06	1.6E-05	1.6

Gene symbol	P-value	FDR	Fold change (log 2)
MRI1	1.9E-13	1.5E-12	-1.7
PKD1	9.4E-15	8.9E-14	-1.7
FOXRED2	3.2E-06	8.3E-06	-1.6

G-set-2- Cohort 1 MM vs. HD

**Gene symbol**	**P-value**	**FDR**	**Fold change (log 2)**
RPS17	1.5E-12	5.3E-10	2.5
RPS16	1.2E-10	3.3E-08	1.9
RPL30	4.7E-09	9.3E-07	2.1
RPL35A	5.8E-09	9.3E-07	2.0
CLIC1	6.2E-09	9.3E-07	2.0
WDR83OS	2.8E-08	3.2E-06	1.9
HNRNPC	4.2E-08	4.0E-06	2.3
SNRPB	6.3E-08	5.1E-06	2.1
DBI	7.0E-08	5.3E-06	2.6
PSMB8	9.8E-08	6.5E-06	2.5
RPS27	1.3E-07	7.8E-06	1.7
IER5	1.9E-07	1.1E-05	2.1
TMSB10	2.0E-07	1.1E-05	2.3
MRPL53	2.1E-07	1.1E-05	2.4
RPS20	2.6E-07	1.3E-05	1.7
PGP	2.8E-07	1.3E-05	1.7
EMC7	3.0E-07	1.3E-05	2.5
UBALD2	3.0E-07	1.3E-05	2.9
RPS12	3.4E-07	1.4E-05	2.3
RPS14	3.6E-07	1.4E-05	1.8
CDKN1A	4.0E-07	1.4E-05	3.3
GNG5	4.8E-07	1.6E-05	2.1
TOMM22	6.6E-07	2.1E-05	1.9
DDT	7.6E-07	2.3E-05	3.1
PLIN3	2.5E-06	6.5E-05	1.9
MPC2	2.9E-06	7.2E-05	2.1
TRIAP1	3.0E-06	7.4E-05	1.5
SLC25A5	4.5E-06	1.0E-04	2.1
CHCHD2	7.0E-06	1.5E-04	2.0
BTF3	1.0E-05	2.1E-04	1.5
RPS5	1.1E-05	2.2E-04	2.8

ARF6	1.3E-05	2.6E-04	1.6
PRELID1	1.3E-05	2.6E-04	1.8
RPLP0	1.6E-05	3.0E-04	2.0
RPL15	1.8E-05	3.3E-04	1.9
MZT2A	1.9E-05	3.4E-04	1.5
RPL18A	2.8E-05	4.6E-04	1.7
LDHA	3.0E-05	4.7E-04	2.3
SNX18	3.0E-05	4.7E-04	1.5
KPNA2	3.1E-05	4.8E-04	2.2
EIF4E2	3.3E-05	5.1E-04	1.6
CCDC85B	3.5E-05	5.2E-04	1.6
AEN	4.0E-05	5.9E-04	1.9
OAZ1	4.3E-05	6.1E-04	1.9
RPL7	5.0E-05	6.9E-04	1.8
RAN	5.2E-05	7.1E-04	1.5
TAGLN2	5.6E-05	7.4E-04	2.2
CLPP	7.3E-05	9.2E-04	1.5
RPSA	7.4E-05	9.2E-04	2.1
RPS15A	1.2E-04	1.3E-03	1.6
RPS18	1.6E-04	1.7E-03	2.1
STOML2	1.7E-04	1.8E-03	1.8
FAM162A	2.0E-04	2.1E-03	2.2
TMEM70	2.5E-04	2.5E-03	1.8
EIF4H	3.4E-04	3.3E-03	1.7
YWHAQ	3.9E-04	3.6E-03	1.6
C19orf53	4.8E-04	4.2E-03	1.5
ANXA2	5.4E-04	4.7E-03	2.1
HNRNPA0	5.4E-04	4.7E-03	1.6
TPI1	5.7E-04	4.8E-03	1.5
TUBB4B	5.8E-04	4.8E-03	1.7
NAMPT	6.3E-04	5.2E-03	2.5
MIF	6.8E-04	5.5E-03	1.6
SNRPC	8.2E-04	6.4E-03	1.6
PMF1	9.5E-04	7.1E-03	1.7
UQCRFS1	1.1E-03	7.9E-03	1.6
COPS2	1.1E-03	8.0E-03	1.9
CYC1	1.2E-03	8.6E-03	1.6

IGLL5	1.5E-24	1.6E-21	-10.2
IGHE	1.2E-13	6.2E-11	-6.1
IRF2	2.5E-08	3.2E-06	-3.3
CCDC117	3.0E-08	3.2E-06	-2.6
SRP54	4.8E-08	4.2E-06	-3.2
NCAPD2	7.9E-08	5.6E-06	-2.7
RB1	3.7E-07	1.4E-05	-4.5
FCMR	3.9E-07	1.4E-05	-2.4
KDM1A	4.2E-07	1.5E-05	-1.9
SF3B3	4.5E-07	1.5E-05	-2.3
IGKV3D-15	6.0E-07	1.9E-05	-7.0
MINPP1	8.7E-07	2.5E-05	-4.1
CD81	1.1E-06	3.1E-05	-3.6
HDAC2	1.4E-06	3.8E-05	-2.5

BAZ1B	1.8E-06	4.9E-05	-1.9
SMC1A	2.5E-06	6.5E-05	-2.2
ATXN10	3.2E-06	7.7E-05	-1.8
GFPT1	4.1E-06	9.6E-05	-2.5
MSH2	4.7E-06	1.1E-04	-2.7
CDC6	6.7E-06	1.5E-04	-2.6
HOOK3	7.5E-06	1.6E-04	-2.3
CKAP5	9.9E-06	2.1E-04	-2.3
APOBEC3C	1.6E-05	3.0E-04	-2.0
SLC35F5	2.2E-05	3.8E-04	-2.9
EXOC5	2.2E-05	3.8E-04	-2.8
CDC23	2.6E-05	4.4E-04	-2.0
CTR9	2.7E-05	4.5E-04	-2.1
DNAJC21	3.0E-05	4.7E-04	-1.9
ELAVL1	3.5E-05	5.2E-04	-1.5
NUP37	4.1E-05	5.9E-04	-2.1
LAX1	4.5E-05	6.3E-04	-3.0
BNIP3	5.1E-05	7.0E-04	-2.4
CPSF2	6.1E-05	8.0E-04	-2.3
RBBP4	6.2E-05	8.0E-04	-2.0
ACAT1	6.8E-05	8.8E-04	-2.2
COPB2	7.1E-05	9.0E-04	-2.5
CAB39	7.5E-05	9.2E-04	-2.0
SLBP	8.7E-05	1.0E-03	-1.6
VPS35	1.0E-04	1.2E-03	-1.9
TOP1	1.1E-04	1.2E-03	-2.0
RBL1	1.2E-04	1.4E-03	-1.8
PRIM1	1.3E-04	1.4E-03	-2.0
SF3A1	1.3E-04	1.4E-03	-1.5
BTN2A2	1.4E-04	1.5E-03	-1.6
CLTC	1.5E-04	1.6E-03	-1.8
PPP2R2A	2.0E-04	2.1E-03	-1.8
DCAF12	2.2E-04	2.2E-03	-1.9
DCPS	2.3E-04	2.4E-03	-1.6
EAF2	2.5E-04	2.5E-03	-2.3
ERO1A	2.8E-04	2.8E-03	-1.8
KDELR2	3.0E-04	2.9E-03	-1.8
PAGR1	3.1E-04	3.0E-03	-1.7
LEO1	3.4E-04	3.2E-03	-1.7
CCNI	3.9E-04	3.6E-03	-1.5
XRCC5	4.3E-04	3.9E-03	-1.7
ARCN1	4.5E-04	4.1E-03	-1.5
CYB5B	4.6E-04	4.1E-03	-1.8
DOCK8	4.7E-04	4.2E-03	-2.0
INPP5D	5.3E-04	4.6E-03	-2.0
API5	5.9E-04	4.9E-03	-1.7
NDUFS1	6.2E-04	5.1E-03	-1.6
RRM1	6.4E-04	5.2E-03	-1.7
MEF2C	6.9E-04	5.5E-03	-2.1
GDI2	7.1E-04	5.6E-03	-1.6
XBP1	7.7E-04	6.1E-03	-1.6
CBX5	8.1E-04	6.3E-03	-2.0

ALDH18A1	8.6E-04	6.6E-03	-2.1
ADD1	9.6E-04	7.1E-03	-1.5
SMC3	9.7E-04	7.2E-03	-1.6
NEMP1	1.0E-03	7.3E-03	-2.2
LTV1	1.1E-03	8.0E-03	-1.8
UCHL5	1.2E-03	8.6E-03	-1.7

G-set-2- Cohort 2 MM vs. HD

Gene symbol	P-value	FDR	Fold change (log 2)
MAGEC1	1.8E-09	1.1E-08	7.1
PRC1	3.5E-18	2.6E-16	4.8
DCTPP1	1.7E-15	5.4E-14	4.5
CBR1	1.0E-06	3.8E-06	4.3
NDC80	1.1E-10	9.1E-10	4.2
LIG1	1.2E-09	7.6E-09	3.6
EXOSC5	9.3E-23	3.1E-20	3.4
CDKN1A	2.4E-19	3.1E-17	3.3
CKS2	2.2E-09	1.4E-08	3.1
CDCA8	2.3E-04	5.3E-04	3.1
NDRG1	1.2E-06	4.3E-06	3.1
AHCY	1.6E-13	3.0E-12	3.0
NUP155	4.3E-21	7.4E-19	3.0
RRM2	1.1E-04	2.8E-04	2.9
CYC1	1.7E-16	7.1E-15	2.9
PSMB8	2.9E-15	8.5E-14	2.9
GGH	6.9E-06	2.3E-05	2.7
AEN	5.9E-19	6.6E-17	2.5
CCDC85B	1.1E-08	6.1E-08	2.4
SSRP1	3.2E-21	6.7E-19	2.4
GYG1	1.6E-16	6.7E-15	2.4
UBL4A	8.1E-13	1.3E-11	2.4
MTHFD1	4.5E-17	2.5E-15	2.4
FBL	3.9E-15	1.1E-13	2.4
NEMP1	8.4E-12	9.9E-11	2.3
OXCT1	4.2E-14	9.0E-13	2.3
UBALD2	6.9E-12	8.4E-11	2.3
LDHB	2.5E-17	1.7E-15	2.3
GPI	3.0E-14	6.7E-13	2.3
HSPA9	1.9E-27	9.8E-25	2.3
MZT2A	3.9E-17	2.4E-15	2.3
RUVBL1	4.2E-09	2.5E-08	2.2
RAN	1.3E-16	6.1E-15	2.2
TUBB4B	6.8E-13	1.2E-11	2.2
NUP62	2.2E-16	8.4E-15	2.2
EID1	1.2E-18	1.1E-16	2.2
HSPB1	5.1E-05	1.4E-04	2.2
C1QBP	2.6E-12	3.5E-11	2.2
C21orf91	1.8E-08	9.1E-08	2.2
CCT5	1.3E-12	2.0E-11	2.1
MCM7	3.9E-13	6.9E-12	2.1
SLC39A8	7.7E-12	9.2E-11	2.1
STOML2	4.6E-12	5.8E-11	2.1

VBP1	1.1E-14	2.6E-13	2.1
MAPKAPK2	1.1E-11	1.2E-10	2.1
EMC7	3.8E-16	1.3E-14	2.1
PSMA4	4.1E-17	2.4E-15	2.1
IDH2	2.4E-06	8.5E-06	2.1
ALDOA	3.2E-15	8.9E-14	2.1
HNRNPAB	1.1E-13	2.1E-12	2.1
SLC25A5	2.0E-11	2.1E-10	2.0
SVIP	1.4E-10	1.2E-09	2.0
LAP3	2.7E-08	1.4E-07	2.0
MCM6	9.3E-08	4.3E-07	2.0
HDGF	9.0E-12	1.1E-10	2.0

IGLL5	1.2E-22	3.1E-20	-7.2
IGHE	2.0E-32	2.1E-29	-6.2
CD68	9.7E-09	5.4E-08	-4.2
NRGN	5.2E-11	5.0E-10	-4.1
CD81	9.8E-14	2.0E-12	-3.8
TNFRSF1B	8.0E-08	3.7E-07	-3.2
DEK	2.1E-06	7.6E-06	-2.5
CLU	1.5E-18	1.3E-16	-2.4
BNIP3	8.4E-09	4.8E-08	-2.2
INCENP	6.4E-07	2.5E-06	-2.1
LEFTY2	4.1E-09	2.5E-08	-1.9
EMP2	1.8E-11	2.0E-10	-1.9
MB	1.4E-08	7.7E-08	-1.9
KIF20B	2.1E-07	9.2E-07	-1.8
PLK1	8.3E-06	2.7E-05	-1.8
UBE2S	5.4E-10	3.8E-09	-1.8
LCP2	3.8E-06	1.3E-05	-1.8
C12orf75	4.5E-07	1.8E-06	-1.7
TMSB4X	3.3E-05	9.4E-05	-1.7
SLC43A3	5.0E-07	2.0E-06	-1.7
GPC4	6.6E-09	3.8E-08	-1.7
EMID1	2.8E-11	2.8E-10	-1.7
EEF1A2	7.9E-10	5.4E-09	-1.7
ARHGAP11A	6.4E-05	1.7E-04	-1.7
CHAF1B	6.9E-04	1.5E-03	-1.6
ZMIZ1	5.5E-06	1.8E-05	-1.6
HJURP	1.4E-04	3.5E-04	-1.6
RAP1A	1.6E-10	1.3E-09	-1.6
ARL6IP1	4.3E-11	4.2E-10	-1.6
ITGB1	3.0E-05	8.5E-05	-1.6
UBASH3B	4.8E-06	1.6E-05	-1.6
PKMYT1	3.2E-07	1.3E-06	-1.5
MTPN	4.4E-05	1.2E-04	-1.5
TCF19	6.7E-06	2.2E-05	-1.5
HELZ2	7.3E-06	2.4E-05	-1.5

Table S4 12 gene signature- G-set-1

CLPP	caseinolytic mitochondrial matrix peptidase proteolytic subunit
ZNF593	zinc finger protein 593
SLC25A5	solute carrier family 25 member 5
SNRPB	small nuclear ribonucleoprotein polypeptides B and B1
POP7	POP7 homolog, ribonuclease P/MRP subunit
SEC61B	Sec61 translocon beta subunit
NDUFB8	NADH:ubiquinone oxidoreductase subunit B8
MAPKAPK2	mitogen-activated protein kinase-activated protein kinase 2
UBL5	ubiquitin like 5
UQCRFS1	ubiquinol-cytochrome c reductase, Rieske iron-sulfur polypeptide 1
NDUFA6	NADH:ubiquinone oxidoreductase subunit A6
CTPS1	CTP synthase 1

77 gene signature-G-set-2

Gene symb	Gene Name
CLPP	caseinolytic mitochondrial matrix peptidase proteolytic subunit
CYC1	cytochrome c1
CDKN1A	cyclin dependent kinase inhibitor 1A
GGH	gamma-glutamyl hydrolase
GPI	glucose-6-phosphate isomerase
HIGD1A	HIG1 hypoxia inducible domain family member 1A
CKS2	CDC28 protein kinase regulatory subunit 2
RAN	RAN, member RAS oncogene family
AEN	apoptosis enhancing nuclease
TUBB4B	tubulin beta 4B class IVb
EMC7	ER membrane protein complex subunit 7
RPL35A	ribosomal protein L35a
FXR1	FMR1 autosomal homolog 1
NDRG1	N-myc downstream regulated 1
PMVK	phosphomevalonate kinase
PRELID1	PRELI domain containing 1
TOMM22	translocase of outer mitochondrial membrane 22
SLC25A5	solute carrier family 25 member 5
CLIC1	chloride intracellular channel 1
FBL	fibrillarin
TRIAP1	TP53 regulated inhibitor of apoptosis 1
MAPKAPK2	mitogen-activated protein kinase-activated protein kinase 2
ATRAID	all-trans retinoic acid induced differentiation factor
C19orf53	chromosome 19 open reading frame 53
ANXA2	annexin A2
EIF4A3	eukaryotic translation initiation factor 4A3
GYG1	glycogenin 1
CFL1	cofilin 1
EEF1E1	eukaryotic translation elongation factor 1 epsilon 1
BTF3	basic transcription factor 3
PSMA2	proteasome subunit alpha 2
HPRT1	hypoxanthine phosphoribosyltransferase 1
BTG1	BTG anti-proliferation factor 1
RTN4	reticulon 4
MIF	macrophage migration inhibitory factor
SNX18	sorting nexin 18

AHCY	adenosylhomocysteinase
PSMB8	proteasome subunit beta 8
CYSTM1	cysteine rich transmembrane module containing 1
CCT8	chaperonin containing TCP1 subunit 8
WDR83OS	WD repeat domain 83 opposite strand
OCIAD2	OCIA domain containing 2
UCK2	uridine-cytidine kinase 2
RCC1	regulator of chromosome condensation 1
MZT2A	mitotic spindle organizing protein 2A
RPSA	ribosomal protein SA
UBALD2	UBA like domain containing 2
CHST11	carbohydrate sulfotransferase 11
DBI	diazepam binding inhibitor, acyl-CoA binding protein
SVIP	small VCP interacting protein
UQCRFS1	ubiquinol-cytochrome c reductase, Rieske iron-sulfur polypeptide 1
PGP	phosphoglycolate phosphatase
MAGEC1	MAGE family member C1
LDHA	lactate dehydrogenase A
SNRPB	small nuclear ribonucleoprotein polypeptides B and B1
PSMD14	proteasome 26S subunit, non-ATPase 14
NDUFB6	NADH:ubiquinone oxidoreductase subunit B6
LDHB	lactate dehydrogenase B
HMCES	5-hydroxymethylcytosine
HNRNPC	heterogeneous nuclear ribonucleoprotein C
LAP3	leucine aminopeptidase 3
CNN2	calponin 2
PLIN3	perilipin 3
RSL24D1	ribosomal L24 domain containing 1
FAM162A	family with sequence similarity 162 member A
HSPB1	heat shock protein family B
SLC39A8	solute carrier family 39 member 8
STOML2	stomatin like 2
RPS27L	ribosomal protein S27 like
RPL15	ribosomal protein L15
CYTIP	cytohesin 1 interacting protein
HDGF	hepatoma-derived growth factor
HSP90AB1	heat shock protein 90 alpha family class B member 1
KPNA2	karyopherin subunit alpha 2
CCDC85B	coiled-coil domain containing 85B
TPI1	triosephosphate isomerase 1
PRPS1	phosphoribosyl pyrophosphate synthetase 1
